# Numerical simulation study on microfluidic particle separation based on magnetophoretic force

**DOI:** 10.1039/d6ra04721a

**Published:** 2026-08-18

**Authors:** Xiaoxiang Xi, Junbo Yang, Yonghui Yang, Peng Yu, Xue-Bo Chen

**Affiliations:** a School of Electronic and Information Engineering, University of Science and Technology Liaoning Anshan 114051 China xuebochen@126.com; b School of Intelligent Equipment Engineer, Wuxi Taihu University Wuxi 214064 China

## Abstract

Rapid blood sample pretreatment is critical for early bloodstream infection diagnosis, yet direct pathogen extraction is hindered by scarce target microbes and abundant blood cell interference. This work develops a 3D magnetophoretic microfluidic separator to continuously isolate magnetically tagged white blood cells from unlabeled pathogens. Its core innovation integrates periodically grooved microchannels with aligned permanent magnets to amplify transverse magnetic gradients and boost lateral particle deflection. A COMSOL multiphysics model coupling laminar flow, magnetic fields and particle tracking is built to analyze flow, magnetic force and particle motion. Under inlet velocity 75 µm s^−1^ and remanence 0.02 T, non-magnetic particles attain 97.04% separation yield at the target outlet, while magnetic particles drift toward high-gradient zones under dominant magnetophoretic force. Separation efficiency varies non-monotonically with particle size and density, peaking at 83.56% for 4 µm, 1800 kg m^−3^ particles. Cuboid magnets outperform conical and spherical counterparts. This grooved channel-magnet array setup strengthens magnetophoretic deflection and offers a feasible scheme for microfluidic blood pretreatment and pathogen enrichment.

## Abstract 2

A grooved microchannel coupled with aligned permanent magnets amplifies transverse magnetic gradients to enhance magnetophoretic lateral migration and realize high-efficiency magnetic particle separation in microfluidic systems.

## Graphical abstract

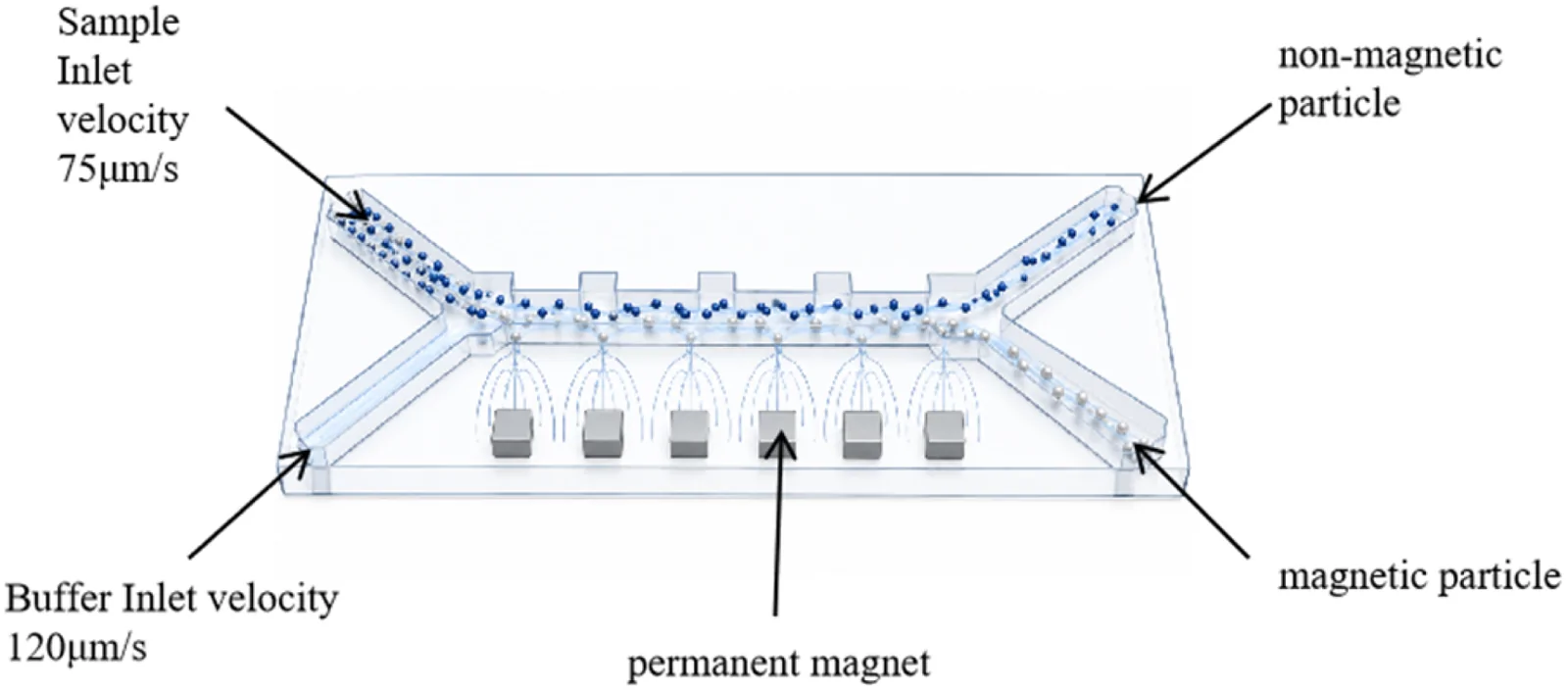

## 1 Introduction

Sepsis is a life-threatening syndrome caused by a dysregulated host response to infection and remains a major challenge in emergency and critical care medicine. The Sepsis-3 consensus provides a widely accepted definition and clinical assessment framework for sepsis and septic shock.^1^ Early identification of the causative pathogen is important for selecting appropriate antimicrobial therapy and improving clinical outcomes. Current clinical guidelines therefore emphasize the timely collection of microbiological samples and the early administration of appropriate antimicrobial treatment. Blood culture remains the reference method for diagnosing bloodstream infections.^2^ In clinical microbiology, blood-culture-based diagnosis is still regarded as a central approach for confirming bacteraemia.^3^ However, culture-based workflows generally require 16–48 h for initial detection. When pathogen identification and antimicrobial susceptibility testing are included, the diagnostic turnaround time can be further prolonged.^4^ Direct pathogen detection is also difficult because the bacterial concentration in bloodstream infections may be as low as 1–10 CFU mL^−1^.^5^ The low abundance of circulating pathogens is accompanied by a much larger background population of erythrocytes, plasma proteins, and other interfering components. Consequently, the performance of rapid molecular and phenotypic assays often depends on an effective sample-preparation step that retains the microorganisms while removing or reducing the blood-cell background.^6^ Previous work has shown that bacteria can be isolated from whole blood using microfluidic approaches.^7^ Elasto-inertial microfluidics has also been used to separate bacteria from whole blood under conditions relevant to sepsis diagnostics.^8^ Nevertheless, bacterial recovery, blood-cell depletion, processing time, and sample volume remain difficult to optimize simultaneously. Microfluidic systems are well suited to this task because they provide controlled transport over short length scales.^9^ They also require relatively small sample volumes and can integrate separation with subsequent detection and analysis.^10^ These advantages make microfluidic separation an important sample-preparation strategy for biological particles and cells.^11^ Particle and cell separation in microchannels can be achieved through passive hydrodynamic mechanisms or through externally applied electric, acoustic, optical, and magnetic fields.^11^ Continuous separation of cells and particles has therefore become a major direction in microfluidic systems.^12^ Among these approaches, magnetophoresis is attractive for biological separation because magnetic forces can be applied remotely through common chip materials and can selectively act on magnetically responsive targets.^13^ The force can be introduced by exploiting the intrinsic magnetic properties of a particle. More commonly, functionalized magnetic micro- or nanoparticles are attached to selected cells, microorganisms, or biomolecules to generate a controllable magnetic response.^14^ Magnetic nanoparticles have been widely investigated for biomedical applications, including separation and targeting.^15^ Permanent magnets, electromagnetic conductors, have all been used to generate the non-uniform magnetic fields required for continuous particle deflection.^16^ In immunomagnetic separation, surface-functionalized magnetic beads bind to target cells through specific antibodies, ligands, or other recognition molecules.^14^ Once labelled, the target cells acquire a magnetic response that can be adjusted independently of their original size and density. Magnetically assisted platforms have been developed for blood-related separation and purification applications.^17^ Microfluidic devices have also been used to isolate bacteria from whole blood for sepsis-related diagnostic workflows. These studies demonstrate the potential of magnetic and microfluidic separation for sample preparation. At the same time, they indicate that practical performance depends on labelling efficiency, magnetic-particle properties, blood composition, flow rate, channel geometry, and magnetic-field distribution.

The trajectory of a magnetically labelled particle is primarily determined by the competition between magnetophoretic force and hydrodynamic drag. Analytical models have shown that the magnetic force acting on a particle depends strongly on the magnetic-field magnitude and field gradient.^18^ For blood-cell-scale magnetic separation, the balance between magnetic force and viscous drag is particularly important.^19^ The magnetophoretic force depends on the magnetic volume and magnetization of the particle–bead complex, as well as on the magnitude and spatial gradient of the magnetic field. The drag force depends on fluid viscosity, particle size, and the relative velocity between the particle and the carrier fluid. Efficient separation therefore requires sufficient transverse magnetic migration during the finite residence time of the particle in the separation region. Channel geometry, flow conditions, magnet arrangement, and particle properties must consequently be examined as a coupled system rather than as independent design variables.^20^ Several recent studies have investigated new magnetic-field sources and microchannel configurations for improving magnetophoretic separation. Asgharivaskasi *et al.* introduced current-carrying plates to produce spatially controlled magnetic-field gradients.^21^ Khashan *et al.*^22^ combined positive and negative magnetophoresis for multi-target particle sorting and examined the influence of magnetic arrangements on particle trajectories. Soika *et al.* compared simplified two- and three-dimensional descriptions of continuous-flow magnetophoresis with a finite-element model.^23^ Their results showed that flow-profile assumptions and the method used to calculate separation efficiency can substantially affect the predicted performance. More recently, Nameni *et al.*^24^ studied a grooved current-carrying conductor and optimized its geometry for magnetic particle separation. These studies confirm that three-dimensional effects and geometric details are important. They also show that magnetophoretic microfluidic separation is already a well-developed field. Thus, the novelty of a numerical design should be demonstrated by a focused comparison of its geometry, field-source configuration, operating conditions, and performance metrics.

## 2 Theoretical concepts

This section presents the governing equations for the coupled magnetic, flow, and particle transport phenomena. The magnetophoretic separation relies on the motion of magnetic particles under magnetic and hydrodynamic forces. Accordingly, we describe the magnetic field model, the magnetophoretic force expression, and the particle dynamics model, which form the theoretical basis for the subsequent numerical simulations and performance evaluation. The magnetophoretic separation process involves the coupled effects of magnetic field, fluid flow, and particle motion. In the present model, magnetic particles are subjected to magnetophoretic force, hydrodynamic drag, gravity, and buoyancy, whereas non-magnetic particles are mainly transported by the background flow. The governing equations used in this work are based on classical magnetophoretic theory, low-Reynolds-number microfluidic flow, and recent magnetophoretic microfluidic separation studies.^24^

In this study, the magnetic field is generated by external permanent magnets. Since the magnetic field does not vary with time and no free electric current exists in the computational domain, the magnetic field can be described using the magnetostatic formulation.^25^ Under magnetostatic conditions, the magnetic flux density satisfies Gauss's law for magnetism:

1 ∇ × *B* = 0

In the equation, *B* represents the magnetic flux density vector. This equation indicates that there are no magnetic monopoles in nature, and magnetic field lines always form closed loops, which is an important physical law that must be satisfied in the analysis of magnetic fields. For linear isotropic magnetic media, the magnetic flux density and magnetic field strength satisfy

2 *B* = *µ*_0_(*H* + *M*)

Here, *H* represents the magnetic field intensity, *M* represents the magnetization intensity, and *µ*_0_ represents the vacuum permeability. For regions without free currents, the magnetic field also satisfies

3 ∇ × *H* = 0

A curl-free vector field can be expressed as the gradient of a scalar potential. Therefore, a magnetic scalar potential function *ψ*_m_ can be introduced, and the magnetic field strength can be expressed as:

4 *H* = −∇*ψ*_m_

Substituting eqn (4) into (2), and using eqn (1) yields the governing equation for the magnetic scalar potential:

5 ∇ × (*µ*_0_∇*ψ*_m_) = 0

By solving this equation, the spatial distribution of the magnetic field intensity inside the microfluidic device can be obtained, thereby providing an external field basis for the calculation of the magnetophoretic force. In a nonuniform magnetic field, particles experience induced magnetization owing to the susceptibility contrast with the medium, resulting in a magnetophoretic force that drives their migration. This driving force generated by the magnetic field gradient is called the magnetophoretic force. The magnetophoretic force is the core driving force for separating magnetic particles from non-magnetic particles, and its magnitude and direction directly determine the displacement behavior of the particles in the microfluidic channel. According to the force relationship of the magnetic dipole in the magnetic field, the force received by the magnetic dipole can be expressed as

6 *F* = ∇(*m* × *B*)

Here, *m* represents the magnetic dipole moment. When the magnetic dipole moment does not change with the spatial position, the above equation can be written as

7 *F* = (*m* × ∇)*B*

For uniformly isotropic spherical particles, their magnetic dipole moment can be expressed as

8 *m* = *V*_p_*M* = *V*_p_Δ*χH*

Here, *V*_p_ represents the volume of the particles. Under the influence of the magnetic field, the particles undergo induced magnetization, and their magnetization intensity can be expressed as

9 *M* = *χ*_p_*H*

Taking into account the difference in magnetic susceptibility between the particles and the surrounding fluid, the expression for the magnetic dipole moment can be obtained.

10 *m* = *V*_p_(*χ*_p_ − *χ*_f_)*H*

where *χ*_p_ and *χ*_f_ represent the magnetic susceptibilities of the particles and the fluid respectively. In an electromagnetic medium, for spherical particles in a linear magnetic medium under the action of a uniform magnetic field, their equivalent magnetic dipole moment can be expressed as11
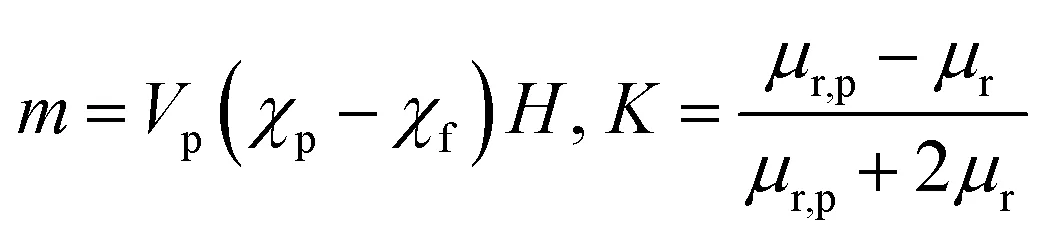
Here, *K* is the Clausius–Mossotti type magnetization coefficient, describing the influence of the difference in magnetic permeability between the particle and the medium on the magnetization process. *µ*_r,p_ and *µ*_r_ are the relative permeabilities of the particle and fluid, respectively. Substituting eqn (11) into the magnetic dipole force expression and combining it with the relationship between magnetic flux density and magnetic field strength *B* = *µ*_0_*H*, the expression for the magnetophoretic force is obtained:

12 *F*_m_ = *µ*_0_*V*_p_*K*(*H* × ∇)*H*

Further utilizing the vector identity (*H* × ∇)*H* = ½∇(*H*^2^), the commonly used expression for the magnetophoretic force is derived:

13 *F*_m_ = ½*µ*_0_*V*_p_*K*∇(*H*^2^)

This formula indicates that the magnitude of the magnetophoretic force depends not only on the particle volume and susceptibility difference but is also closely related to the spatial gradient of the magnetic field strength. Therefore, in microfluidic magnetic separation devices, optimizing the magnet structure to enhance the magnetic field gradient is a key engineering approach to improve separation efficiency.^26^

Within the microfluidic channel, particles moving in the fluid medium also experience a hindering force due to fluid viscosity, known as the hydrodynamic drag force. Since the microchannel dimensions are on the micrometer scale, the fluid flow Reynolds number is typically low. Under these conditions, inertial effects can be neglected, and fluid motion is primarily governed by viscous forces. Therefore, the fluid flow can be described using an incompressible laminar flow model. The governing equation is the Navier–Stokes equation:^27^

14 *ρ*(*u* × ∇)*u* = −∇*p* + *µ*∇^2^*u*

coupled with the continuity equation:

15 ∇ × *u* = 0

where *ρ* is fluid density, *u* is fluid velocity vector, *p* is pressure, and *µ* is dynamic viscosity.

Under low Reynolds number conditions, the drag force on a particle in the fluid can be described by Stokes' law:^28^

16 *F*_D_ = 3π*µd*_p_(*u* − *v*_p_)

where *d*_p_ is the particle diameter, and *v*_p_ is the particle velocity. Additionally, the particle is subject to the combined action of gravity and buoyancy in the fluid. The net force can be expressed as:

17 *F*_g_ = (*ρ*_p_ − *ρ*_f_)*V*_p_*g*

where *ρ*_p_ and *ρ*_f_ are the particle and fluid densities, respectively, and *g* is the gravitational acceleration vector. Considering the magnetophoretic force, fluid drag force, gravity, buoyancy, and other forces, the motion of a particle under the Lagrangian description framework can be described by Newton's second law:^29^18
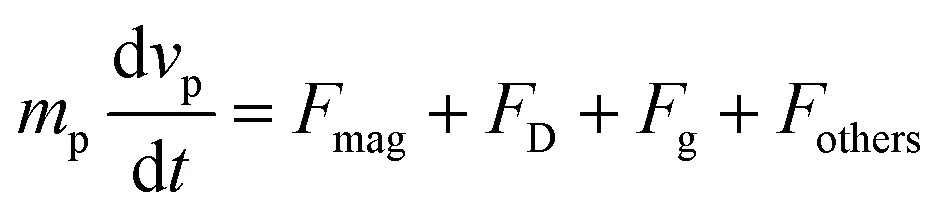


In the numerical simulation process of this paper, the steady-state distributions of the magnetic and flow fields are first solved. These are then used as external field inputs to the particle kinetics model. The particle trajectories within the microchannel are calculated through a one-way coupling approach, thereby achieving continuous separation of magnetic and non-magnetic particles in the microfluidic device.

## 3 Model design

**Fig. 1:**
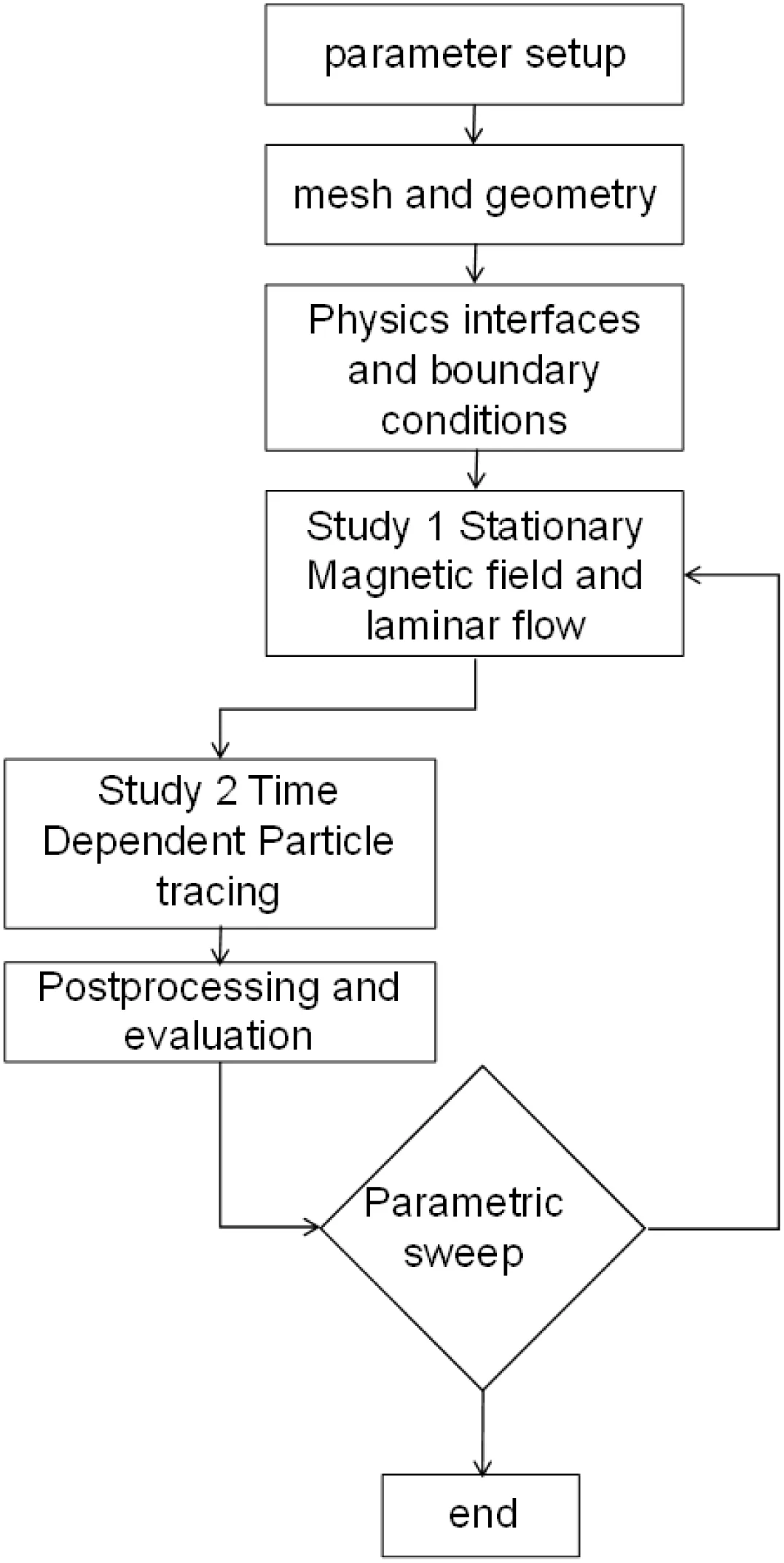
Model solution steps.

This study was based on the multi-physics simulation software COMSOL Multiphysics 6.3, coupling three physics interfaces: laminar flow, magnetic fields (no currents), and particle tracing. The flow and magnetic field distributions were obtained by solving the Navier–Stokes and magnetic scalar potential equations, respectively. In the simulation, it was assumed that the fluid was an incompressible Newtonian fluid and the flow was in a stable laminar state (Fig. 1).

### 3.1 Geometric structure and grid partition of the microfluidic separation model

**Fig. 2:**
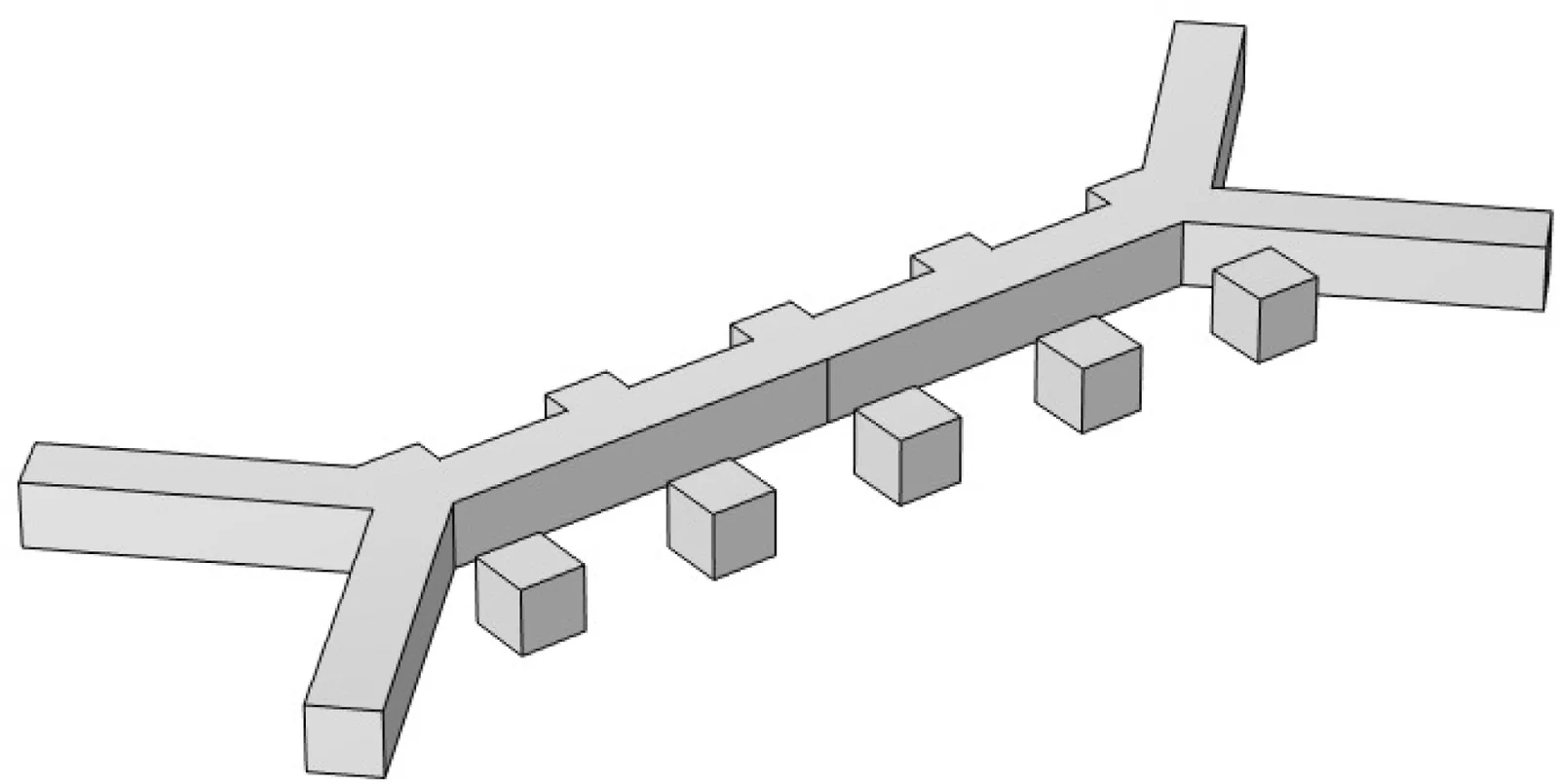
Microfluidic separation model.

The proposed magnetophoretic microfluidic separator was constructed as a three-dimensional model, as shown in Fig. 2. The model consists of a main separation channel, periodic grooves, an external permanent-magnet array, and a surrounding air domain. The main channel has a slender rectangular geometry along the flow direction, with the separation region located in the central part of the channel. The model mainly consists of the main microfluidic separation channel, the upper concave structure, the permanent magnet structure, and the external air domain. The main channel has a slender rectangular structure along the flow direction, with the particle separation area located in the middle of the channel, and entrances and exits are set at both ends to ensure a stable flow state of the fluid before and after entering the separation area. Periodic grooves are introduced along the upper side of the main channel to modify the local flow and magnetic-field distribution, thereby promoting the lateral deflection of magnetically responsive particles.

During the geometric modeling process, the overall size of the channel is at the micrometer scale, and its length is much greater than the height and width of the channel to ensure that the fluid flow is in a low Reynolds number laminar state. The size of the concave structure is at the same order of magnitude as the channel height, and its introduction causes a change in the local cross-section of the microchannel, without significantly increasing the flow resistance, and has a significant impact on the flow field and magnetic field (or electric field) distribution. The external air domain adopts a regular rectangular structure, enclosing the entire microfluidic chip and the magnetic structure inside, to simulate the propagation and attenuation behavior of the external field in the air in the real physical environment.

To improve accuracy and reliability, we applied local mesh refinement in regions with strong field gradients, such as the microchannel, groove edges, and magnet vicinity. The overall computational domain is discretized using unstructured grids, and the grid elements mainly include triangular and tetrahedral elements. For regions where the physical quantities change significantly, such as the interior of the microfluidic channel, the edge of the groove structure, and the vicinity of the permanent magnet, a local meshing method is adopted to improve the ability to depict the gradient distribution of the flow field and magnetic field; for the external air domain and the space far from the separation region, a relatively coarser grid setting is used to balance the calculation accuracy and efficiency. The meshing parameters and specific values for each region are shown in Table 1.

**Table 1:** Grid parameters

Name	Max element size	Min element size	Maximum growth rate	Curvature factor	Narrow-region resolution
Microfluidic channel	21.2	6.32	1.15	0.6	0.7
Air domain and far-field region	31.6	9.49	1.2	0.7	0.6

As can be seen from the figure, the internal grid distribution of the microfluidic channels and groove structures is relatively dense, which can well depict the complex geometric features within the channels and their influence on the flow field and external field distribution; while in the air domain and far-field area, the grid size gradually increases, ensuring the balance between computational efficiency and numerical accuracy.

### 3.2 Mesh independence study

**Fig. 3:**
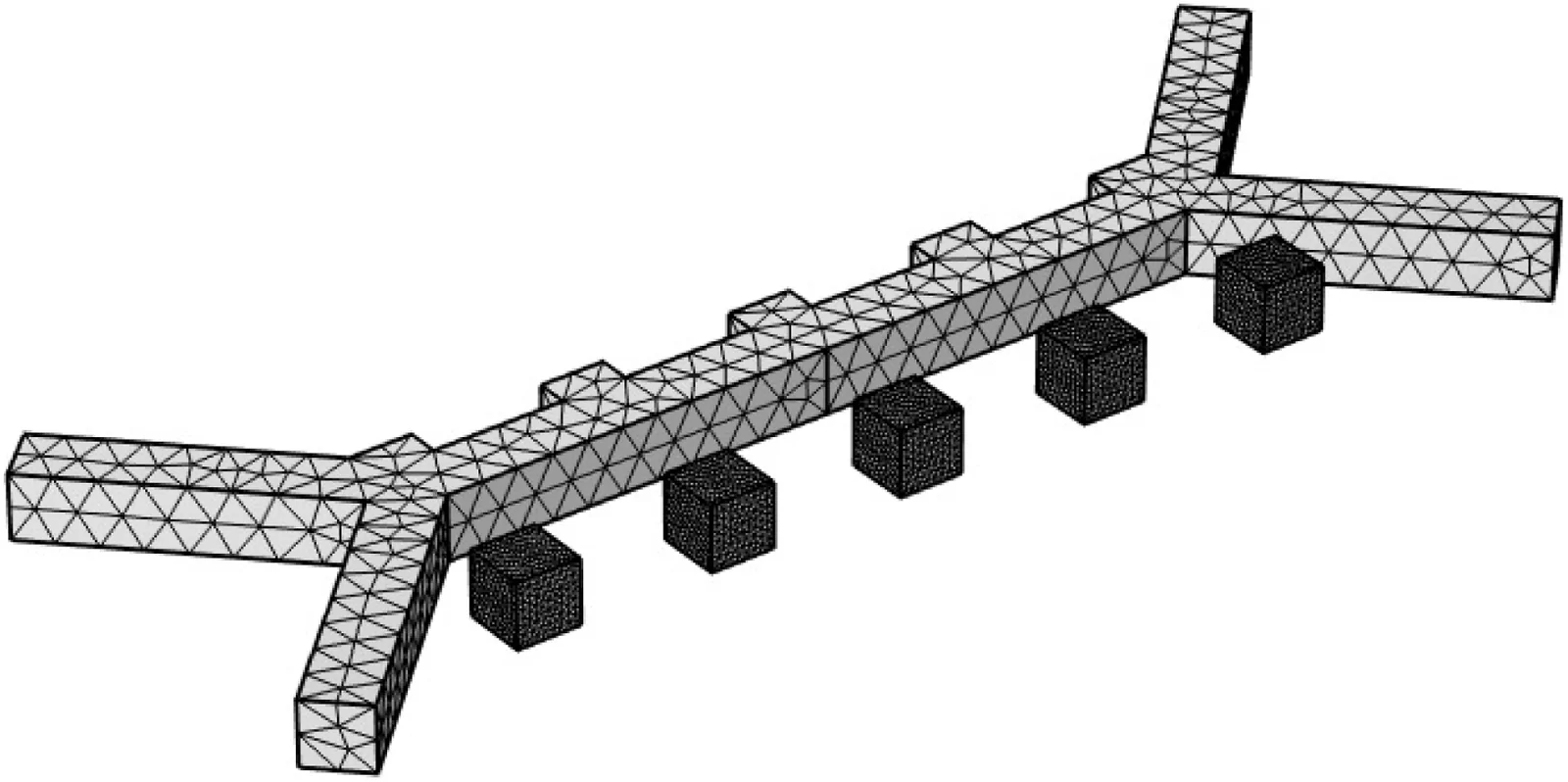
Model mesh.

To ensure the numerical reliability of the magnetophoretic microfluidic separation model, a mesh independence study was performed before the subsequent parametric simulations (Fig. 3).

In the mesh independence analysis, nine mesh levels were tested, including extremely coarse, extra coarse, coarser, coarse, normal, fine, finer, extra fine, and extremely fine meshes. The corresponding number of mesh elements increased from 9232 to 9 388 098. During mesh generation, local refinement was mainly applied in the microfluidic channel, groove edges, channel-wall region, and the vicinity of the permanent magnets, where large velocity gradients and magnetic-field gradients were expected. Relatively coarser elements were used in the external air domain and far-field region to reduce the computational cost.

**Fig. 4:**
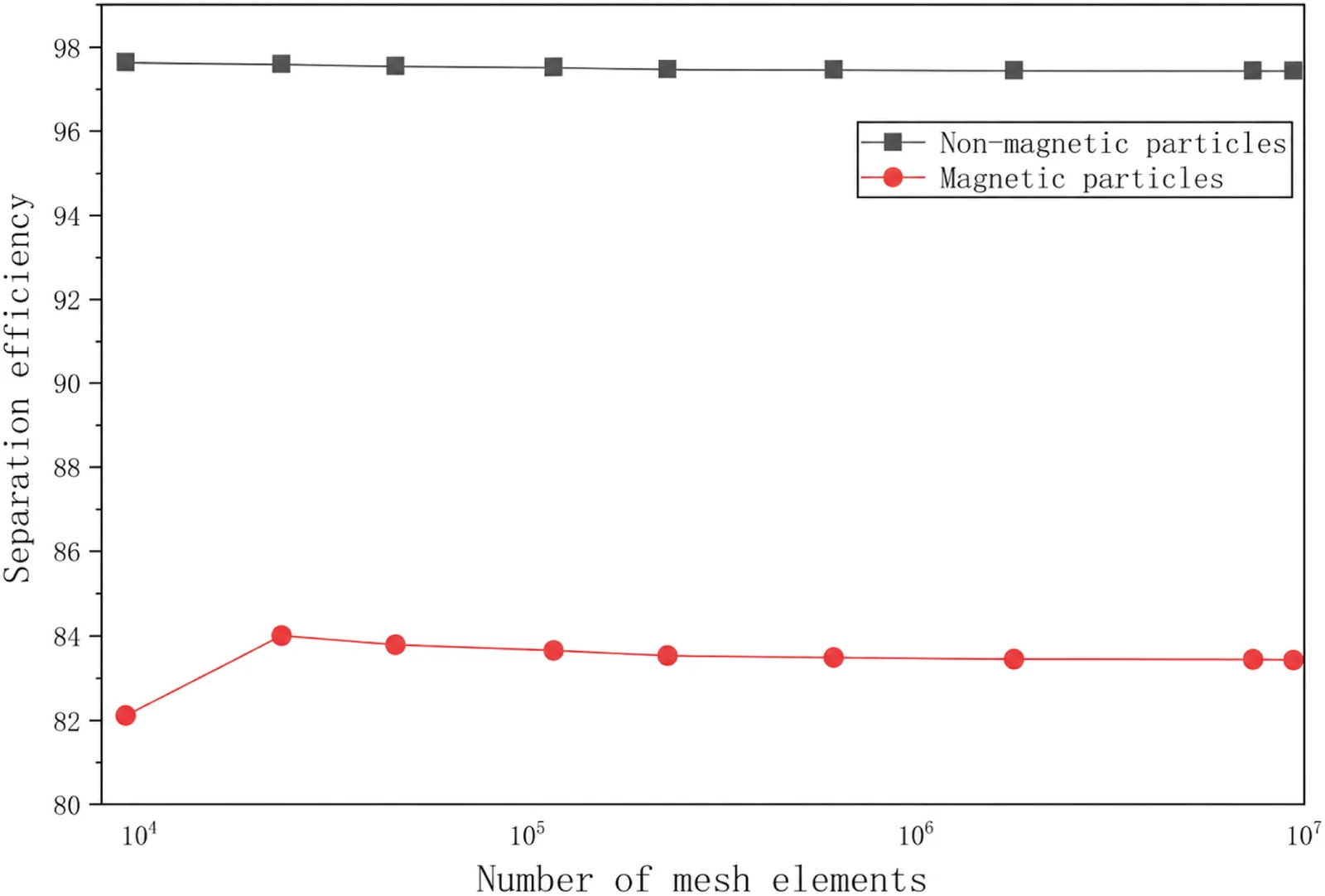
Mesh convergence test.

The mesh independence results are shown in Table 2 and Fig. 4. The separation efficiency of non-magnetic particles remained highly stable for all mesh levels, varying only from 97.62% to 97.42%. This indicates that the predicted motion of non-magnetic particles is not sensitive to mesh refinement, because their trajectories are mainly governed by the stable laminar flow field. In contrast, the separation efficiency of magnetic particles showed a relatively larger variation when the mesh was coarse. When the number of mesh elements increased from 9232 to 23 276, the magnetic-particle separation efficiency changed from 82.13% to 84.33%. This difference indicates that the magnetic-particle trajectories are more sensitive to the spatial resolution of the magnetic-field-gradient region and near-wall region.

**Table 2:** Mesh independence study based on separation efficiency

Mesh level	Number of elements	Separation efficiency of non-magnetic particles (%)	Separation efficiency of magnetic particles (%)
Extremely coarse	9232	97.62%	82.13%
Extra coarse	23 276	97.57%	84.33%
Coarser	45 758	97.53%	83.71%
Coarse	117 023	97.51%	83.69%
Normal	229 601	97.46%	83.56%
Fine	615 843	97.45%	83.52%
Finer	1 792 832	97.43%	83.48%
Extra fine	7 390 489	97.42%	83.47%
Extremely fine	9 388 098	97.42%	83.45%

As the mesh was further refined, the separation efficiencies gradually approached stable values. When the number of mesh elements exceeded 229 601, further mesh refinement caused only negligible changes in the calculated separation efficiencies. Specifically, when the mesh was refined from 229 601 elements to 9 388 098 elements, the separation efficiency of non-magnetic particles changed from 97.46% to 97.42%, while that of magnetic particles changed from 83.56% to 83.45%. The variations were only 0.04 percentage points and 0.11 percentage points, respectively. Therefore, the normal mesh with 229 601 elements was selected for the following simulations, because it provides a reasonable balance between numerical accuracy and computational cost. In addition to mesh refinement, the convergence of the outlet-based separation efficiency with respect to the number of released particles was also examined. Particle numbers of 250, 500, 1000, and 2000 were tested under the reference operating condition. When the particle number exceeded 1000, the change in separation efficiency was below 1%, and 1000 particles were therefore used in the following simulations.

### 3.3 Permanent magnet structure and magnetic field source modeling

To form a non-uniform magnetic field within the microfluidic channel, a permanent magnet array is arranged outside the channel as the magnetic field source. The permanent magnets are arranged regularly along the separation zone direction of the microchannel, and their positions and spacings are designed reasonably to correspond to the upper groove, in order to generate a magnetic field distribution with a large gradient within the channel. The magnetization characteristics of the permanent magnets are described by setting the residual magnetic flux density, thereby generating a stable static magnetic field without introducing free currents. In the magnetic field modeling, the permanent magnet area is described using the magnetic statics model, and the magnetic parameters are set according to the actual material properties. The magnetic potential control equation is solved to obtain the magnetic field intensity distribution of the permanent magnets and the surrounding space, providing necessary magnetic field information for the calculation of magnetophoretic force.

### 3.4 Air domain and infinite element domain setup

In the magnetophoretic microfluidic separation model, the spatial distribution of the magnetic field not only exists within the microfluidic channel and the permanent magnets, but also extends to the external space of the chip and gradually decays. Therefore, when performing numerical calculations of the magnetic field, it is necessary to reasonably describe the propagation behavior of the magnetic field in the external space to avoid non-physical constraints of the calculation domain boundary on the magnetic field distribution. To simulate the distribution of the magnetic field in the air in the real physical environment, an air calculation domain is set up outside the microfluidic channel and the permanent magnet structure. The air domain is a rectangular cuboid that encloses the entire chip and is assigned the relative permeability of air. By introducing the air domain, the magnetic field can freely propagate and close in the external space of the microchannel, thus ensuring the continuity and physical rationality of the magnetic field distribution.

It should be noted that if the external air domain is not introduced in the magnetic field modeling, and only the microfluidic channel and the permanent magnet structure are modeled, the magnetic field will be restricted within a limited calculation domain, and the magnetic field lines are numerically forced to be truncated at the geometric boundary, making it impossible to form a real closed structure. In this case, the magnetic potential distribution tends to be uniform, and the magnetic flux density gradient significantly weakens, making it difficult to reflect the magnetic field characteristics generated by the permanent magnets in the actual physical environment. After introducing the air domain, the magnetic field can propagate freely in the external space of the microchannel and naturally close, and the magnetic field lines can start from the permanent magnets, pass through the microchannel, and form a continuous closed loop in the air, thus truly reflecting the magnetic field distribution law under the magnetic statics condition. Combined with the treatment of the far-field boundary in the infinite element domain, the magnetic field lines gradually decay in the region far from the permanent magnets, effectively avoiding the non-physical constraints of the limited calculation domain boundary on the magnetic field distribution. Since the magnetic field problem is an open boundary problem, the theoretical solution extends infinitely in space. The boundary conditions of the finite calculation domain may affect the magnetic field distribution. To reduce the interference of the calculation domain boundary on the magnetic field results, this paper introduces an infinite element domain on the outer layer of the air domain to simulate the physical process of the magnetic field naturally decaying to zero in the far-field region. Through this method, the reflection or truncation of magnetic field lines at the boundary can be effectively avoided, improving the accuracy and stability of the calculation results of the magnetic field and magnetophoretic force.

## 4 Simulation and result analysis

### 4.1 Boundary conditions and parameter settings

In this paper, boundary conditions and main simulation parameters are uniformly set in each physical field module. In the fluid flow module, the wall surfaces of the microchannels are all set as no-slip boundary conditions. At the inlet, a velocity inlet boundary condition is adopted, and the inlet velocity is set according to the design flow rate; at the outlet, a pressure outlet boundary condition is used, and the pressure is set to the reference pressure value to ensure that the fluid flows stably in a laminar state within the microchannel.

In the magnetic fields, no currents interface, the magnets are modeled by specifying the remanent flux density. A continuous boundary condition is used between the air domain and the infinite element domain to ensure a smooth transition of the magnetic field between different regions. The outer boundary of the infinite element domain is processed by the default far-field method to ensure that the magnetic field naturally decays away from the chip area.

In the fluid flow and particle tracking module, the initial position of the particles is set at the inlet section, and the initial velocity is determined by the local fluid velocity field. When a particle contacts the channel wall, a freeze boundary condition is adopted to avoid non-physical penetration. Such particles are treated as wall-intercepted particles rather than successfully separated particles. The present model includes the no-slip wall effect on the flow field but does not include near-wall hydrodynamic mobility correction for particles moving very close to the wall. The forces acting on the particles, such as magnetophoretic force, fluid drag force, and gravity, are solved according to the aforementioned theoretical models. To facilitate the explanation of the key parameters and their values used in the simulation process, the relevant settings are summarized in Table 3.

**Table 3:** Geometrical design and parameter setting

Parameter	Value	Description
Sample inlet velocity	75 µm s^−1^	Inlet boundary condition
Buffer inlet velocity	120 µm s^−1^	Inlet boundary condition
Fluid dynamic viscosity	3.5 × 10^−3^ Pa s	Fluid property
Remanent flux density	0.02 T	Magnetic field parameter
Relative recoil permeability	1.05	Magnetic field parameter
Main channel length	280 µm	Microchannel length
Main channel width	40 µm	Microchannel width
Main channel depth	40 µm	Microchannel depth
Relative permeability	2000	Particle magnetic property
Magnetic particle diameter	4 µm	Magnetic particle size
Non-magnetic particle diameter	2 µm	Non-magnetic particle size

### 4.2 Model verification and validation

To assess the reliability of the proposed multiphysics model, three numerical verification procedures were conducted. First, the implementation of the magnetophoretic force and hydrodynamic drag was verified using an independent single-particle benchmark problem with an analytical solution. Second, the magnetostatic field calculated by COMSOL Multiphysics was compared with the analytical magnetic field generated by a uniformly magnetized spherical permanent magnet. An independent benchmark model was established to verify the numerical implementation of the magnetophoretic force and Stokes drag force. A spherical magnetic particle was suspended in a Newtonian fluid and subjected to a prescribed constant transverse magnetic-field-squared gradient. In this simplified benchmark, particle–particle interactions, gravitational settling, Brownian motion, and channel-wall effects were excluded so that the magnetophoretic and hydrodynamic force formulations could be evaluated independently. The fluid dynamic viscosity and axial flow velocity were set to

19 *µ* = 3.5 × 10^−3^ Pa s

and *u*_*x*_ = 75 µm s^−1^ respectively, consistent with the operating parameters employed in the complete microfluidic model. A representative magnetic particle diameter of *d*_p_ = 4 µm and a particle density of *ρ*_p_ = 1800 kg m^−3^ were selected based on the parameter range investigated in the particle-separation simulations. The equivalent magnetic polarization coefficient was set to *K* = 0.1. The particle volume was calculated as20
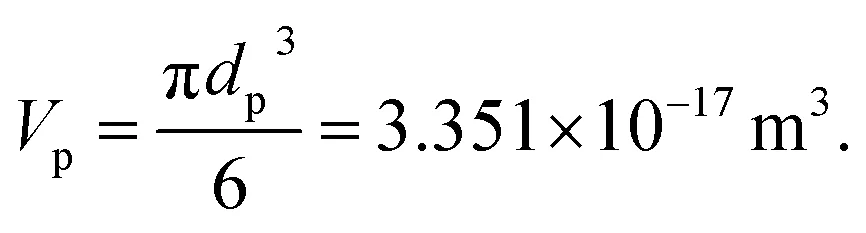


The transverse magnetophoretic force is expressed as21
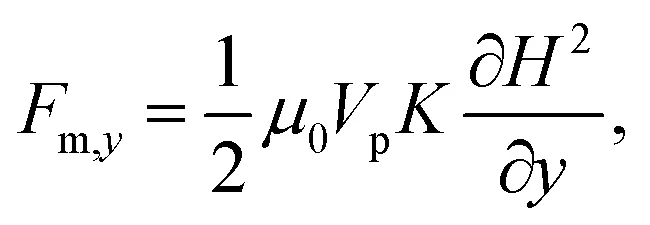
where *µ*_0_ is the vacuum permeability and 
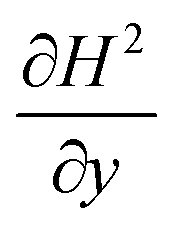
 is the prescribed transverse magnetic-field-squared gradient. Under low particle Reynolds number conditions, the particle motion in the transverse direction is governed by22
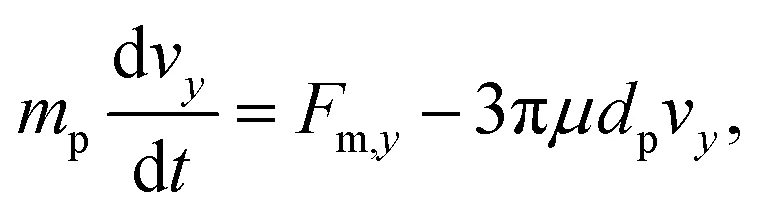
where *v*_*y*_ is the transverse particle velocity and

23 *m*_p_ = *ρ*_p_*V*_p_

is the particle mass. For a particle released with zero initial transverse velocity, the analytical velocity is24
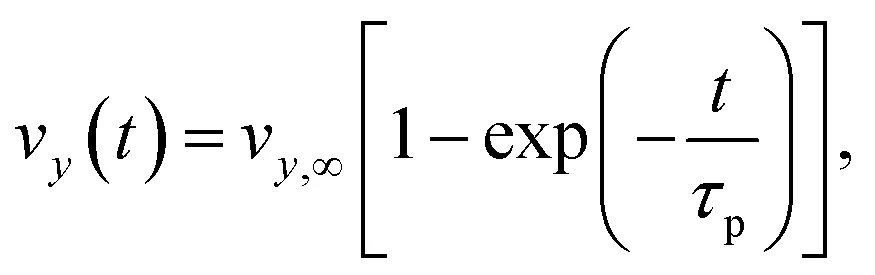
where the terminal magnetophoretic velocity is25
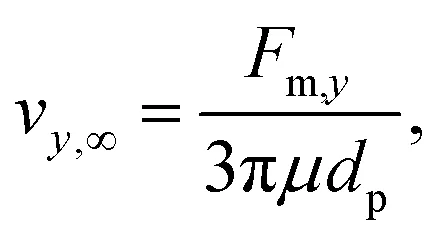
and the particle relaxation time is26
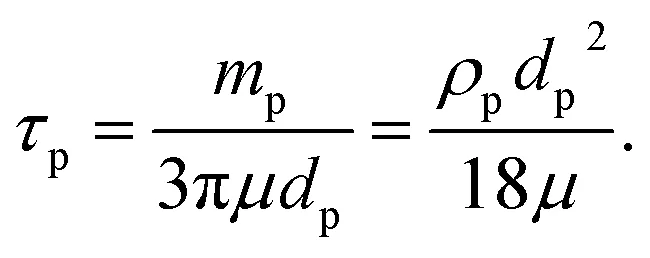


For the selected particle and fluid properties, the particle relaxation time was

27 *τ*_p_ = 4.57 × 10^−7^, s.

Therefore, the particle velocity at

28 *t* = 20*τ*_p_ = 9.14 × 10^−6^, s

was considered equivalent to the terminal velocity because

29 1 − exp(−20) > 0.99999999.

Three representative values of the transverse magnetic-field-squared gradient were examined:30
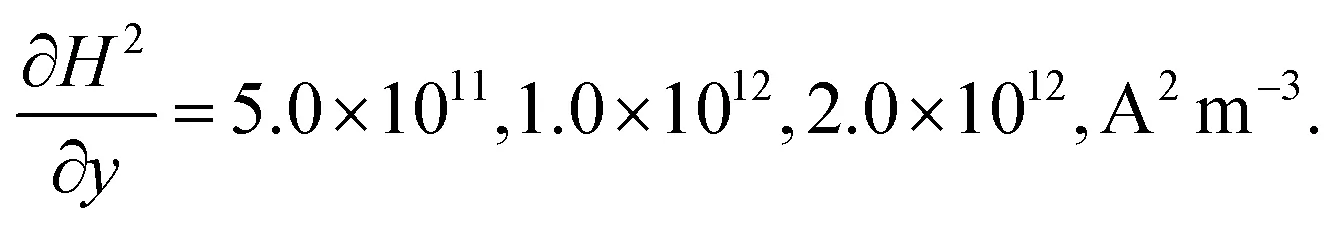


These values covered the low-, intermediate-, and high-gradient conditions encountered in the effective magnetic separation region.

The relative error was calculated as31
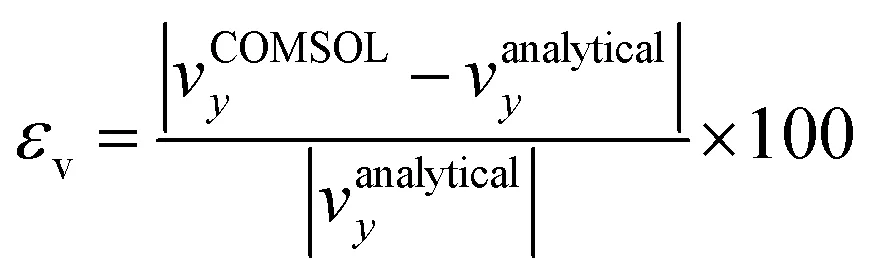


As shown in Table 4, the maximum relative deviation between the COMSOL prediction and the analytical terminal velocity was 6.52%, while the average relative deviation was 5.78%. In addition, both the analytical and numerical results demonstrated an approximately linear relationship between the terminal transverse velocity and the magnetic-field-squared gradient. This agreement confirms that the magnetophoretic force and Stokes drag force were correctly implemented in the particle-tracing model. The analytical transverse displacement of the particle was also calculated as32
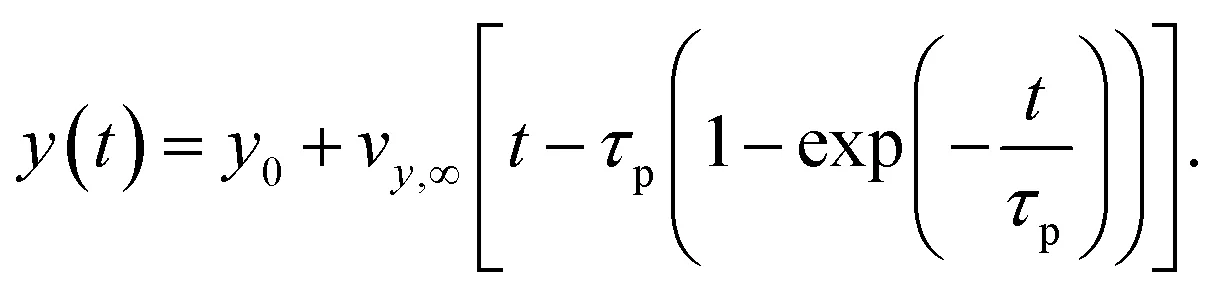


**Table 4:** Comparison between analytical and numerical particle velocities

Case	∂*H*^2^/∂*y* (A^2^ m^−3^)	*F*_m,*y*_ (pN)	Analytical velocity (µm s^−1^)	COMSOL velocity (µm s^−1^)	Relative error
1	5.07 × 10^11^	1.053	7.57	7.93	4.76%
2	1.53 × 10^12^	2.106	14.87	15.84	6.52%
3	2.38 × 10^12^	4.211	29.91	31.72	6.05%

The numerical particle trajectories agreed closely with this analytical displacement equation, further confirming the accuracy of the time-dependent particle-motion calculation. The magnetic field calculation was independently verified using a uniformly magnetized spherical permanent magnet. The spherical geometry was selected because its external magnetic field can be represented analytically, allowing the magnetostatic solution obtained from the magnetic fields, no currents interface to be evaluated without interference from the complex microchannel geometry. The permanent magnet was assigned the same magnetic properties as those used in the complete separation model:

33 *B*_r_ = 0.02 T

and

34 *µ*_r_ = 1.05

where *B*_r_ is the remanent magnetic flux density and *µ*_r_ is the relative recoil permeability.

A spherical magnet with radius *a* was positioned at the center of an air domain. The radius of the surrounding air domain was selected as 8*a*, and an infinite-element domain was added outside the air region to reproduce the natural decay of the magnetic field in an unbounded space. Along the magnetization axis, the analytical magnetic flux density outside the spherical magnet is expressed^30^35
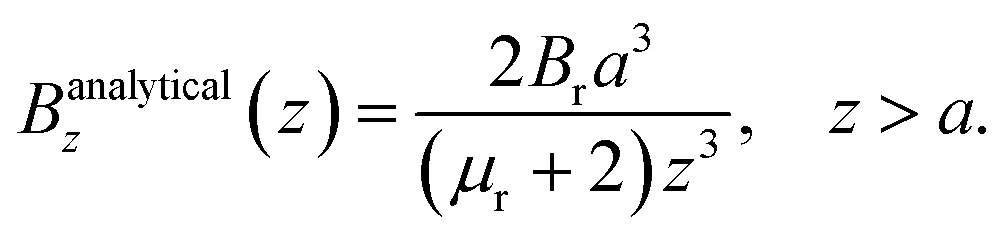


The COMSOL-calculated magnetic flux density was extracted along the magnetization axis over the normalized range36
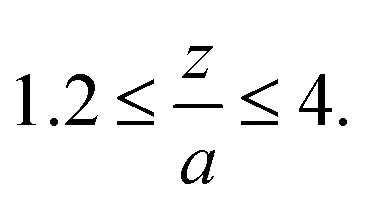


Points immediately adjacent to the magnet surface were excluded because the magnetic-field derivatives are particularly sensitive to mesh resolution at the material interface.

The maximum relative deviation between the analytical and numerical magnetic flux densities was 7.62%. Furthermore, the calculated magnetic flux density exhibited the expected *z*^−3^ decay along the magnetization axis. These results demonstrate that the magnetic material properties, open-boundary treatment, air domain, and infinite-element domain were appropriately defined (Table 5).

**Table 5:** Analytical verification of the magnetic flux density along the spherical–magnet axis

*z*/*a*	Analytical *B*_*z*_ (mT)	Comsol *B*_*z*_ (mT)	Relative error
1.2	7.192	7.538	4.81%
1.5	3.686	3.967	7.62%
2.0	1.639	1.724	5.18%
3.0	0.486	0.512	5.35%
4.0	0.205	0.213	3.90%

Because magnetophoretic separation depends on the magnetic-field gradient rather than solely on the magnetic flux density, the magnetic-field-squared gradient was additionally evaluated using37
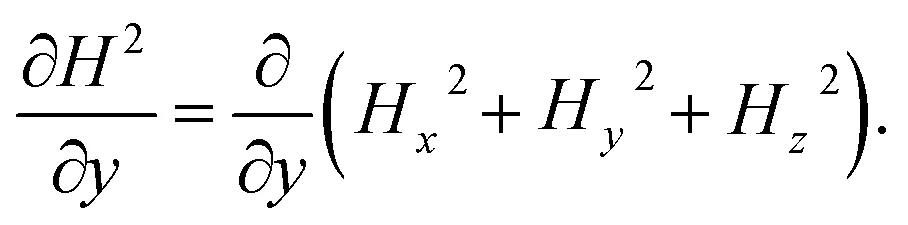


In COMSOL Multiphysics, this quantity was calculated using

38 *d*(mfnc.*H*_*x*_^2^ + mfnc.*H*_*y*_^2^ + mfnc.*H*_*z*_^2^,*y*)

where mfnc denotes the magnetic fields, no currents physics interface. The smooth spatial variation of this quantity outside the magnet and its convergence with mesh refinement confirmed that the magnetic-field gradient used in the particle-force calculation was numerically stable.

### 4.3 Analysis of flow field and magnetic field

**Fig. 5:**
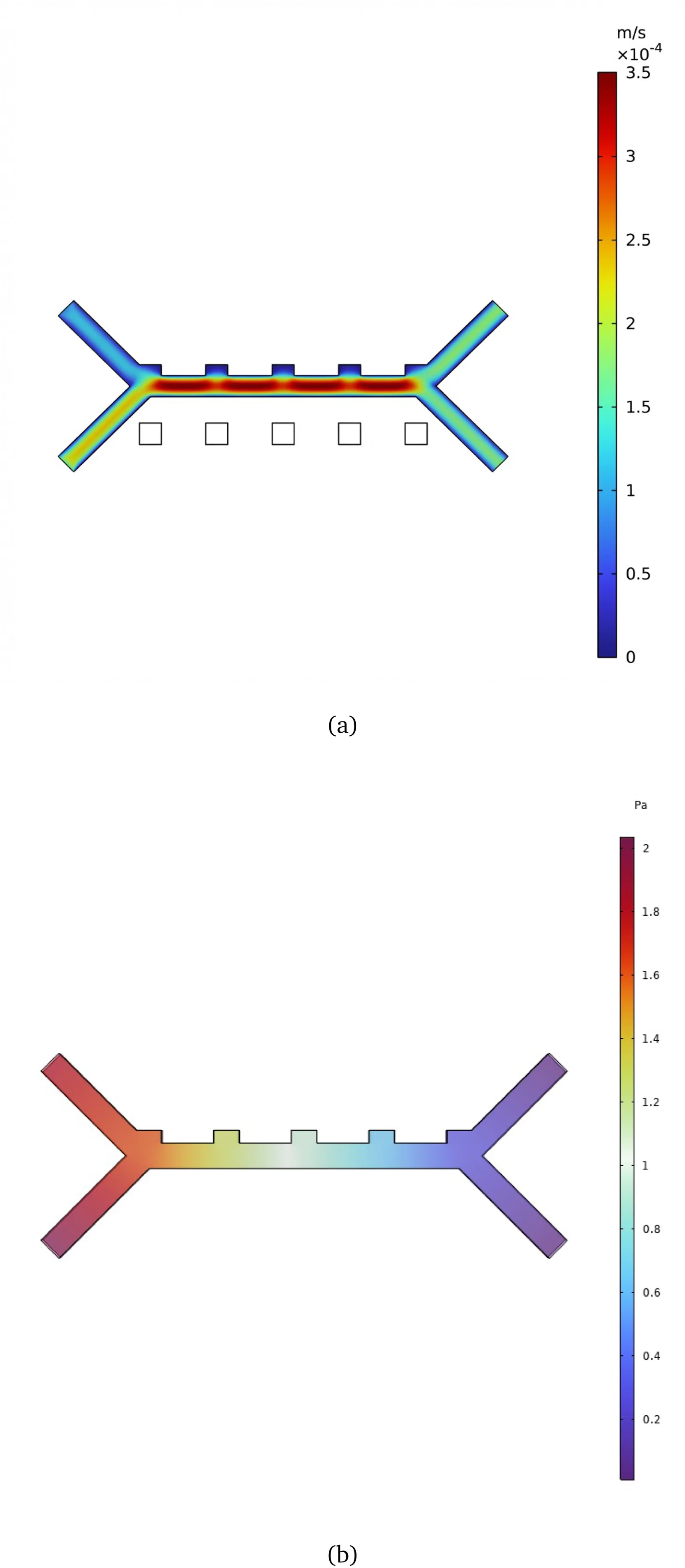
Simulation of flow field and pressure. (a) Velocity distribution. (b) Pressure distribution.

To comprehensively analyze the distribution characteristics of each physical field in the magnetophoretic microfluidic separation model, this section conducts numerical simulation and analysis of the flow field within the microchannel and the applied magnetic field, focusing on the characteristics of the velocity field, pressure field, magnetic flux density distribution, and magnetic potential distribution. The simulation results of the relevant physical fields are shown in Fig. 5.

First, the velocity field distribution within the microfluidic channel is analyzed. As shown in Fig. 5(a), the fluid velocity within the channel exhibits a typical laminar distribution feature: along the main separation channel axis, the velocity distribution changes relatively smoothly; the flow velocity is the highest in the central area of the channel, while it rapidly decays to zero near the wall. In the geometric transition zone between the inlet and outlet, the local flow field shows slight adjustments, but the overall flow remains continuous and stable, and no obvious vortex or reflux phenomena are observed. The distribution of the concave structure within the main channel causes local disturbances to the flow field, forming a limited gradient area of velocity near the concave, but this disturbance does not disrupt the overall laminar structure; instead, it provides a stable background flow field environment for the controlled deflection of the fluid under the magnetic field effect.

Correspondingly, Fig. 5(b) presents the pressure field distribution cloud map within the microchannel. It can be clearly seen that the fluid pressure along the main flow direction shows a monotonically decreasing trend from the inlet to the outlet, which conforms to the basic characteristics of fluid pressure drop along the flow path under laminar flow conditions. In the separation channel area, the pressure isaline distribution is smooth and continuous, and no obvious pressure sudden change or local high-pressure area is observed. This indicates that the introduction of the concave structure does not significantly increase the flow resistance in the channel, thus ensuring the stability and repeatability of the magnetophoretic separation process operation.

**Fig. 6:**
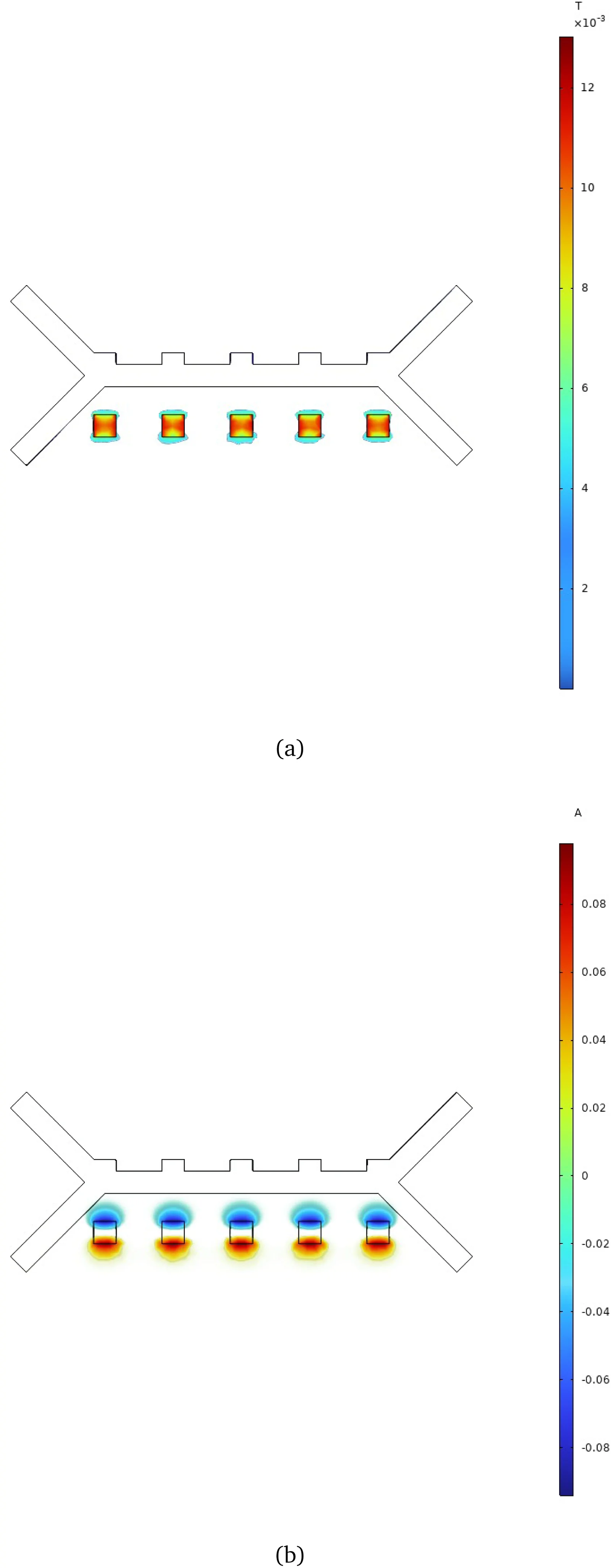
Simulation of magnetic flux density and magnetic scalar potential. (a) Magnetic flux density distribution. (b) Magnetic scalar potential distribution.

In terms of the analysis of magnetic field characteristics, Fig. 6(a) and (b) respectively present the magnetic flux density distribution and magnetic potential distribution within the calculation domain. From the magnetic flux density distribution map (Fig. 6(a)), it can be seen that the magnetic flux density undergoes a drastic change near the permanent magnet, indicating that the magnetic gradient in this area is large; while in the area far from the permanent magnet, the magnetic potential distribution gradually becomes uniform. The microfluidic channel is precisely located in the effective magnetic field action area above the permanent magnet, providing favorable conditions for the controlled deviation of particles in the stable flow field. Further analysis in combination with the magnetic potential distribution map (Fig. 6(b)) reveals that the magnetic potential distribution within the channel is highly corresponding to the structure of the permanent magnet array. In the area above the permanent magnet, a significant high magnetic flux density concentration area is formed, and there is a clear spatial gradient change along the channel transverse direction. This stable and well-gradient magnetic field distribution can effectively generate continuous magnetophoretic force.

### 4.4 Particle trajectory analysis

To further reveal the movement behavior of particles within microfluidic channels under the influence of magnetic fields, this section compares and analyzes the movement trajectories of particles under different conditions based on the numerical results of particle tracking. The focus is on discussing the movement characteristics of particles in the absence of magnetic fields, the condition where only magnetic particles exist, and the condition where magnetic and non-magnetic particles coexist.

**Fig. 7:**
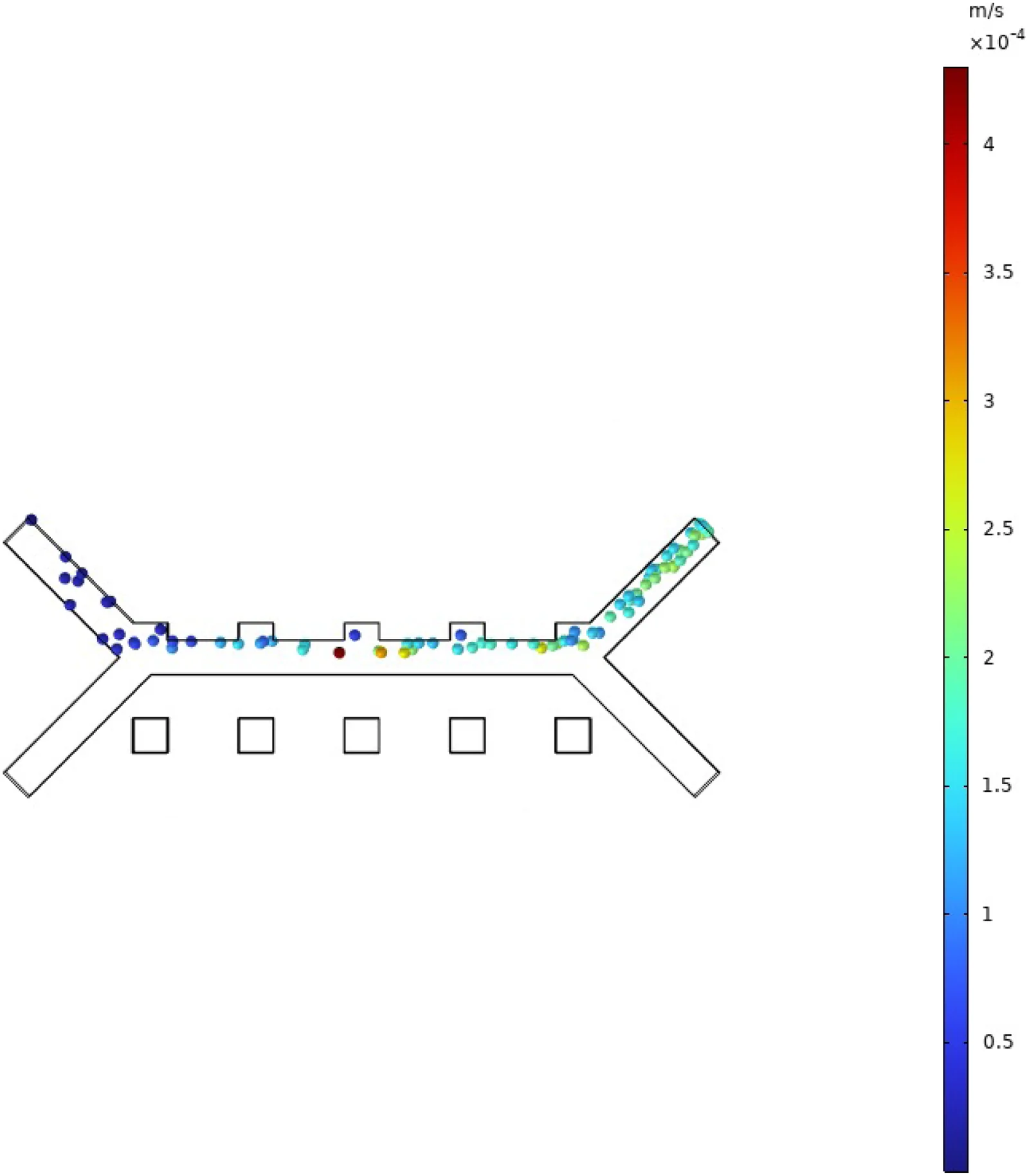
The distribution of the motion trajectories of non-magnetic particles under the condition of no external magnetic field.

First, we simulate particle trajectories without a magnetic field (Fig. 7). In this case, particles are driven only by the fluid drag and follow streamlines without lateral deflection. The particles essentially follow the original streamlines and exhibit no noticeable lateral migration, even through the grooved regions. Finally, they flow out along with the fluid from the corresponding outlet. This result indicates that in the absence of magnetic fields, the geometric structure itself is not sufficient to cause effective particle separation, and the magnetic field effect is the key factor for particle deviation and separation.

**Fig. 8:**
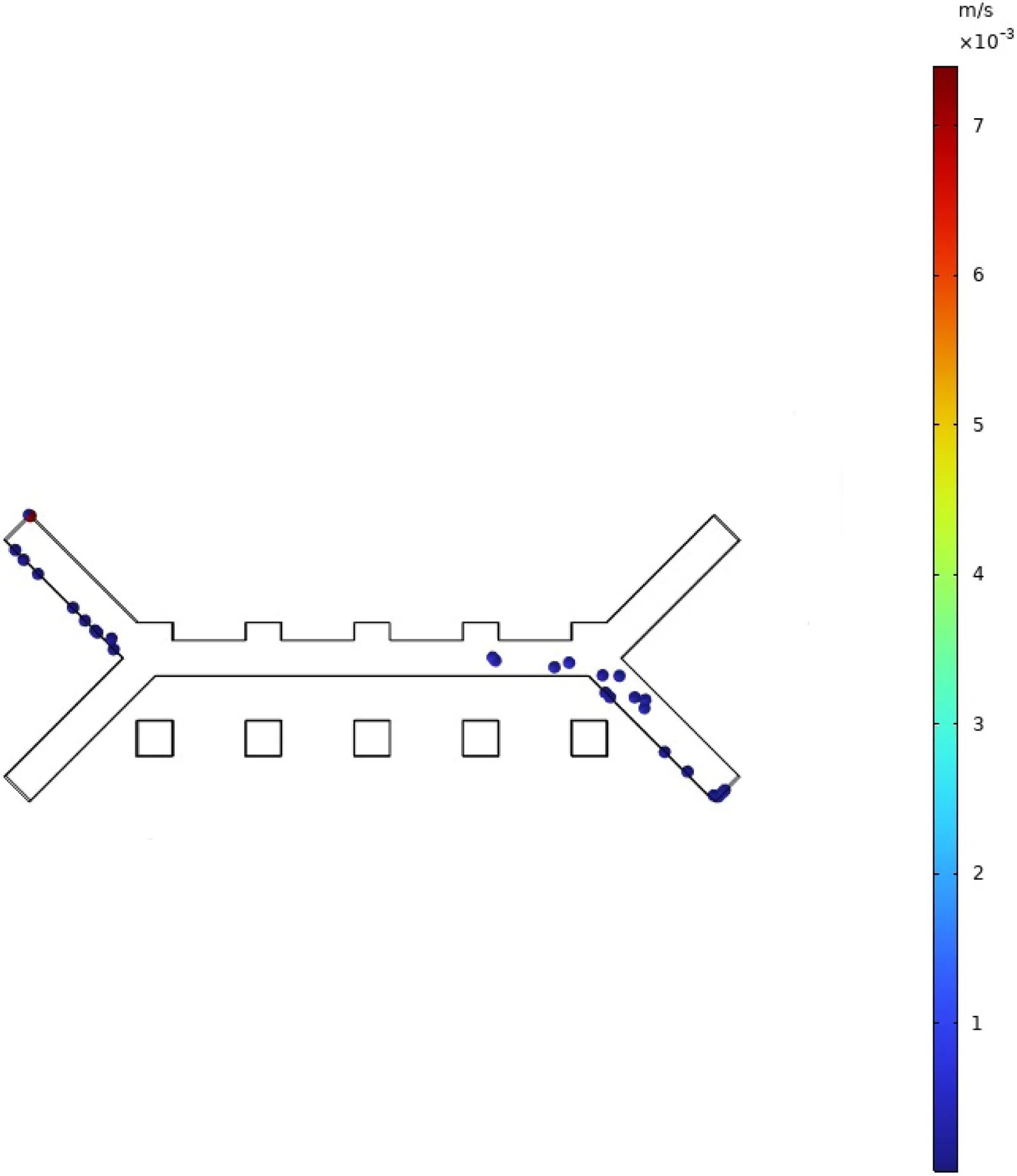
The deflection trajectory of a single magnetic particle under the influence of a magnetic field and a flow field.

When only magnetic particles are introduced, the particles move along the microchannel under the combined action of fluid drag force and magnetophoretic force. The trajectory of the particles is shown in Fig. 8. After entering the main separation channel, the magnetic particles are affected by the non-uniform magnetic field and gradually deviate from the original streamline, and undergo lateral migration towards the region with a larger magnetic gradient. When passing through the area with the distribution of the concave structure, the deviation of the particles further increases, and finally, they are guided to the specific branch channel to exit at the outlet area. This result indicates that under the influence of the non-uniform magnetic field, the magnetophoretic force acting on the magnetic particles gradually increases, and they undergo lateral migration under the competition between fluid drag force and magnetophoretic force. When the magnetophoretic force reaches a comparable magnitude to the fluid drag force in the lateral direction, the particles will gradually deviate from the original streamline, thereby achieving stable and controllable trajectory deviation.

**Fig. 9:**
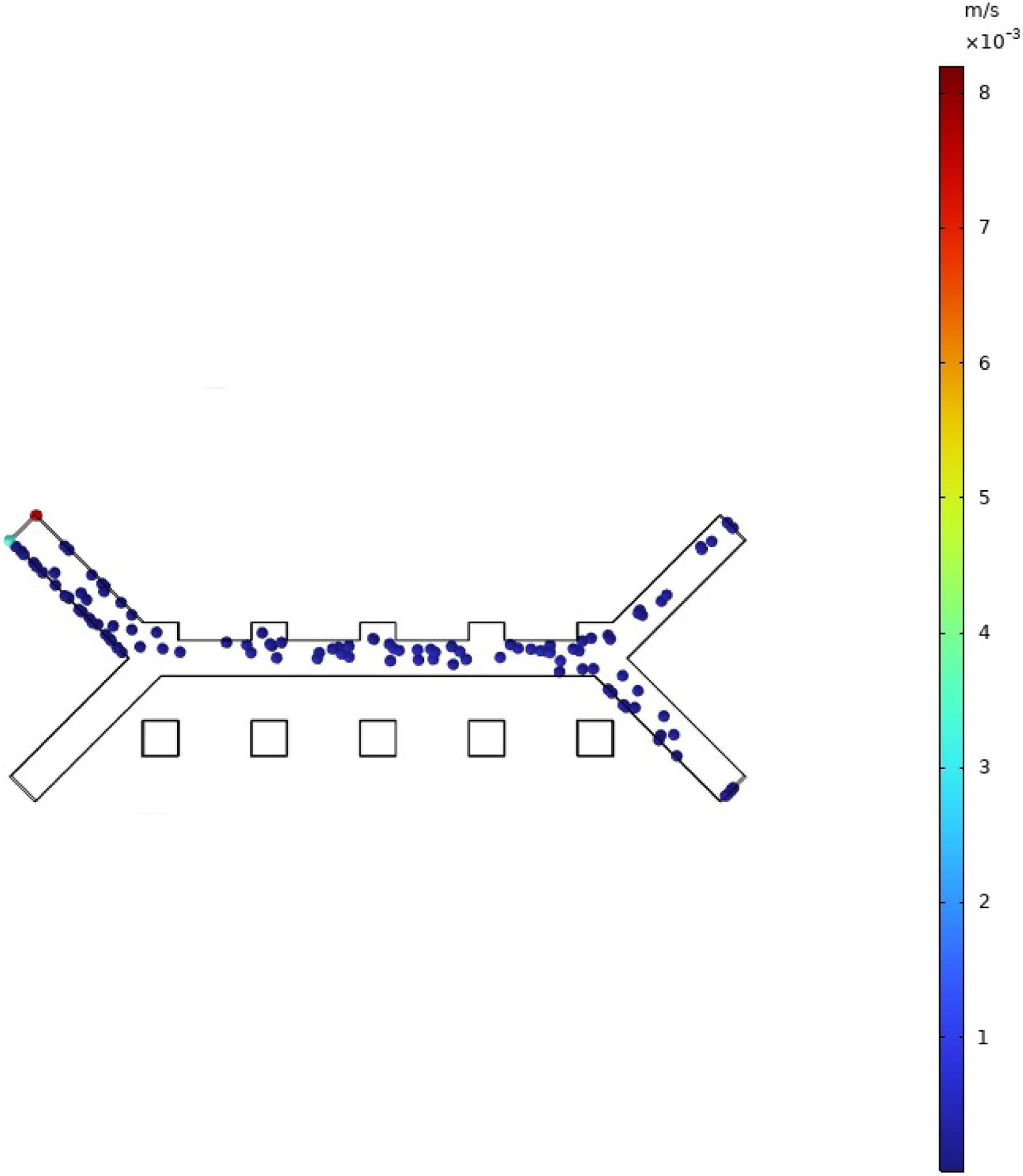
The spatial separation trajectories of magnetic and non-magnetic particles when they coexist.

When both magnetic particles and non-magnetic particles are introduced simultaneously, the particles exhibit significantly different movement behaviors within the microfluidic channel, as shown in Fig. 9. It can be observed that the magnetic particles gradually migrate towards the regions with a larger magnetic field gradient under the influence of the non-uniform magnetic field, their movement trajectories deviate significantly, and eventually flow out from the target outlet; while the non-magnetic particles are mainly controlled by the fluid drag force and basically move along the original flow lines, without significant deviation. The two types of particles gradually form spatial separation in the main separation channel, indicating that this microfluidic magnetophoretic separation model can effectively distinguish particles with different magnetic response characteristics and achieve selective separation.

**Fig. 10:**
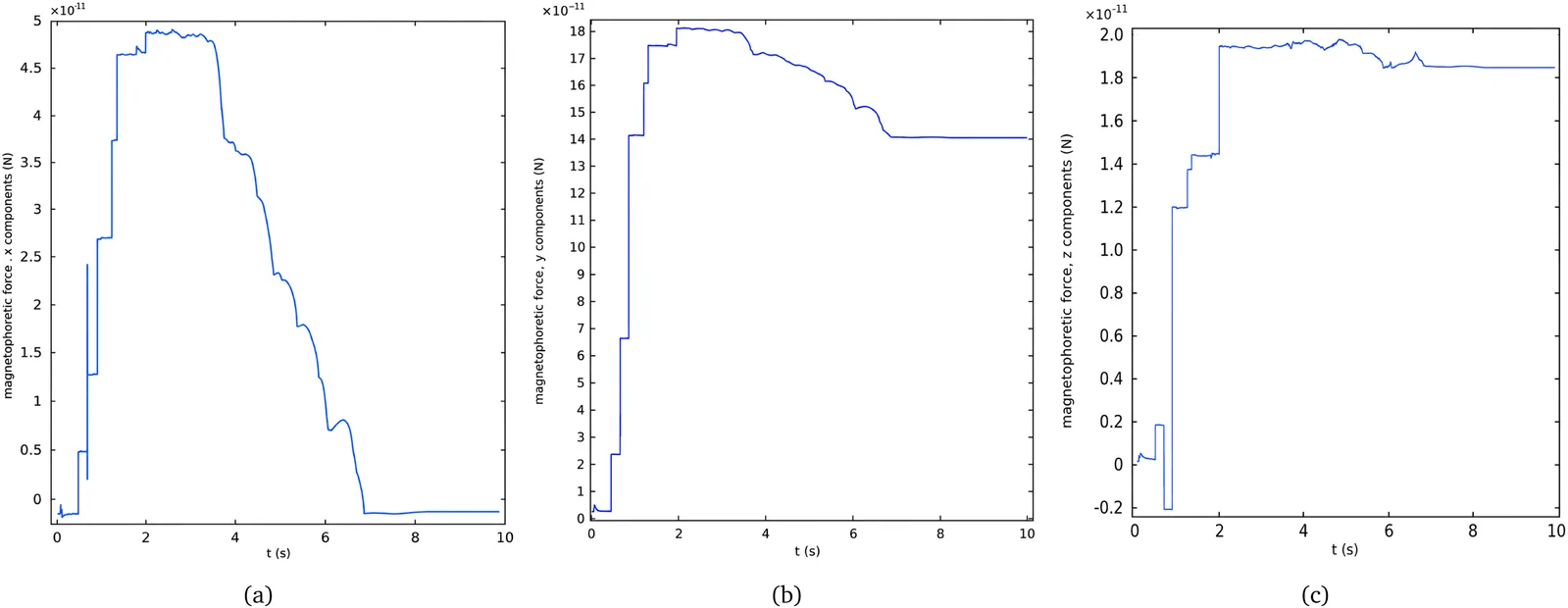
Magnetophoretic force exerted on magnetic particles in the *X*, *Y*, and *Z* directions. (a) *X* direction. (b) *Y* direction. (c) *Z* direction.

From the time curve of the force components (Fig. 10), it can be seen that after the particles enter the effective magnetic field region, the magnetic force they receive rapidly increases (the peak occurs at 2–3 seconds). By analyzing the force conditions in all directions, it is found that the *Y*-component of the magnetic drift force that drives the particles to lateral displacement is much larger than the *X* and *Z* components. It plays a decisive role in breaking the balance of the fluid drag force. This microscopic-scale mechanical change law explains the deflection phenomenon of the particles' macroscopic trajectory and verifies the rationality of the current non-uniform magnetic field design.

The comparative analysis of the particle motion trajectories under different working conditions reveals that the introduction of the magnetic field is the decisive factor for achieving the lateral displacement and separation of particles. The local external field gradient formed by the groove structure further enhances the displacement effect of magnetic particles, laying the foundation for the subsequent quantitative analysis of separation efficiency and performance.

### 4.5 Analysis of separation effect and efficiency

Based on the aforementioned particle trajectory analysis, in order to further quantitatively evaluate the separation performance of the microfluidic magnetophoretic separation model, this paper introduces separation efficiency as an evaluation indicator and analyzes the separation effect of particles under different parameter conditions. The separation efficiency is defined as the ratio of the number of target particles that finally flow out from the target outlet to the total number of such particles released from the inlet.

Under the current model structure and magnetic field parameter conditions, non-magnetic particles are mainly controlled by the fluid drag force, and their trajectory is basically distributed along the main flow direction, which enables them to flow out from the corresponding outlet stably. The separation efficiency of non-magnetic particles is calculated to be 97.04%, indicating that the designed microfluidic structure has a good guiding and separation ability for non-magnetic particles. However, for magnetic particles, although they can undergo a certain degree of lateral displacement under the influence of the magnetic field, there is still room for further improvement in their separation efficiency. Therefore, this paper conducts a systematic analysis of the key parameters that affect the separation efficiency of magnetic particles through parameterized scanning.

Fig. 12 shows the effect of magnetic-particle density on separation efficiency. The separation efficiency first increases and then decreases with increasing particle density. It should be emphasized that, under the present linear magnetization model, particle density does not directly enhance the magnetophoretic force, which is mainly determined by particle volume, magnetic susceptibility contrast, and the magnetic-field-squared gradient. Instead, density affects particle transport through the mass term, gravity–buoyancy balance, and particle relaxation time.

At relatively low density, the particles respond rapidly to the combined action of fluid drag and magnetophoretic force, but their lateral migration may still be insufficient within the finite residence time. As the density increases to an appropriate range, particle trajectories become more favorable for migration toward the target outlet. However, at higher density, gravity–buoyancy effects, finite residence time, outlet allocation, and possible near-wall interception become more influential. Because the estimated Stokes number is very small, the decrease in separation efficiency should not be attributed mainly to particle inertia. Instead, it is more appropriately interpreted as a transport-sensitivity effect under the adopted outlet geometry and wall-boundary conditions.

When the magnetic particle density increases to a certain range, the separation efficiency reaches its maximum value. At this point, a relatively ideal balance state is achieved between the magnetophoretic force and the fluid drag force, and the particles can stably shift to the target outlet. However, as the particle density further increases, the separation efficiency shows a downward trend. This is mainly due to the fact that when the density of magnetic particles is too high, their inertial effect increases, and the stability of the particle movement in the channel decreases, resulting in some particles not effectively entering the target branch channel, thereby reducing the overall separation efficiency.

The dependence of separation efficiency on particle diameter is also non-monotonic (Fig. 13). For small particles, the magnetophoretic force is limited because it scales with particle volume, and the lateral migration within the finite residence time is insufficient. As the particle diameter increases, the ratio between magnetophoretic force and viscous drag increases, leading to stronger transverse deflection and improved outlet allocation. However, when the particle diameter becomes too large, the finite channel height and width, the outlet-dividing streamline, and the wall-contact boundary condition become more important. Larger particles have a higher probability of approaching or contacting the channel wall and may be counted as wall-intercepted or incorrectly allocated particles. Therefore, the decrease in separation efficiency at larger diameters is attributed mainly to geometric confinement, wall interception and outlet allocation effects, rather than to particle inertia alone.

### 4.6 Magnet shape influence analysis

**Fig. 11:**
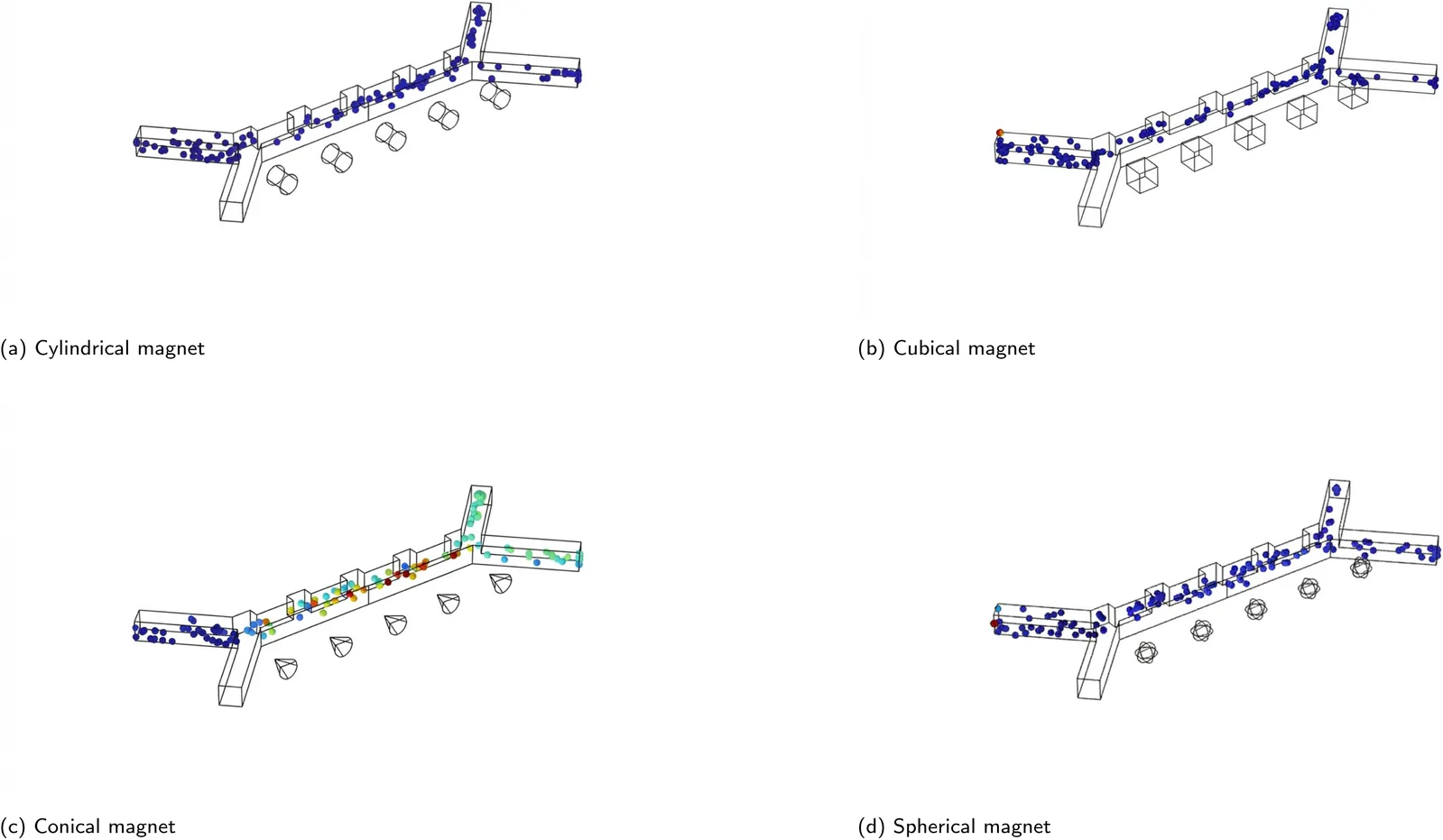
Separation trajectories of magnetic particles under different magnet shapes. (a) Cylindrical magnet, (b) cubical magnet, (c) conical magnet, and (d) spherical magnet.

The influence of magnet geometry was further investigated under fixed magnet material, volume, and remanent flux density. Four magnet shapes, namely cylindrical, cuboid, conical, and spherical magnets, were compared, as shown in Fig. 11. The separation efficiency of non-magnetic particles remained high and changed only slightly among the different magnet shapes, because their trajectories were mainly governed by hydrodynamic drag rather than the magnetic-field distribution.

**Fig. 12:**
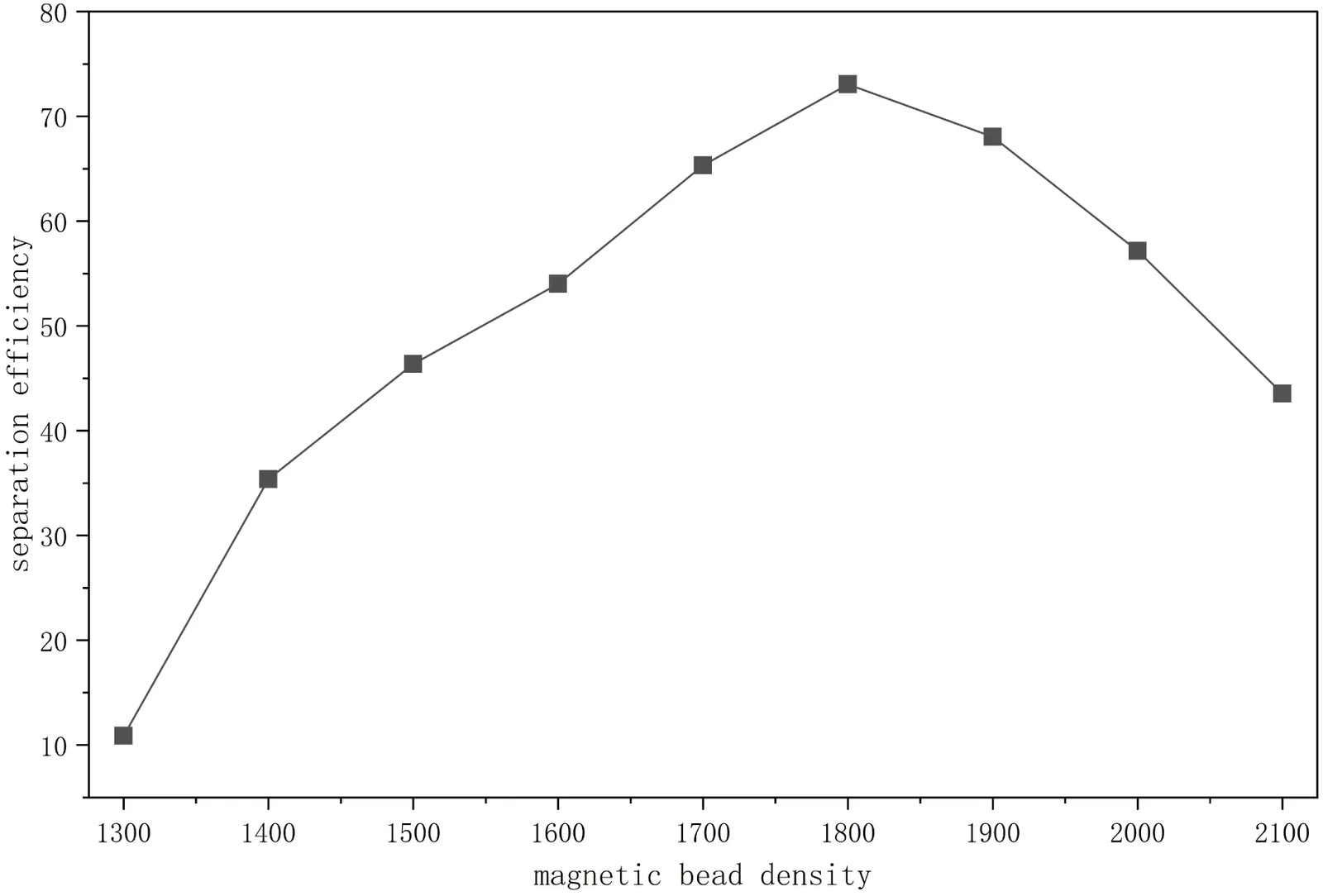
Effect of magnetic bead density on separation efficiency.

**Fig. 13:**
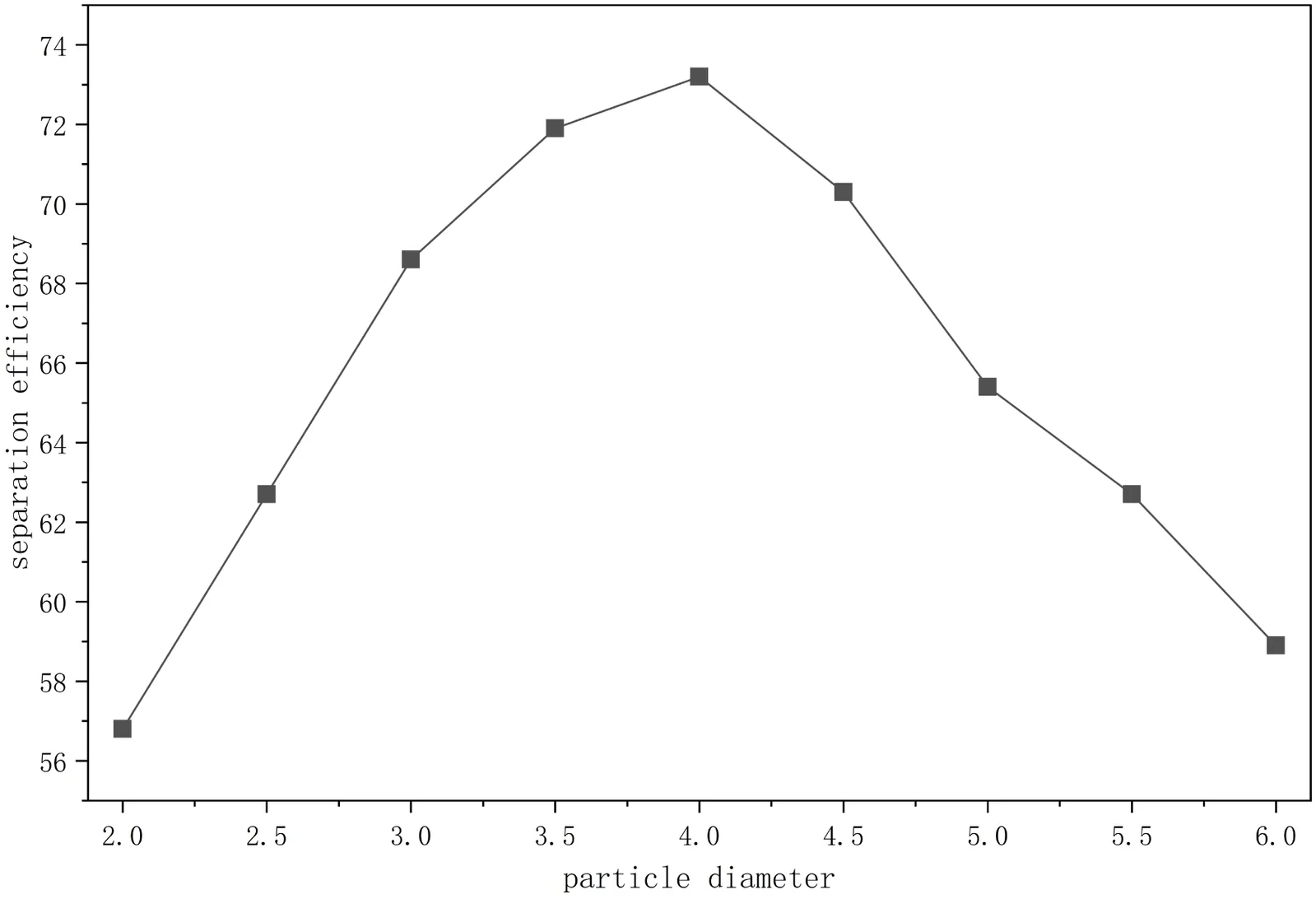
Effect of particle diameter on separation efficiency.

**Fig. 14:**
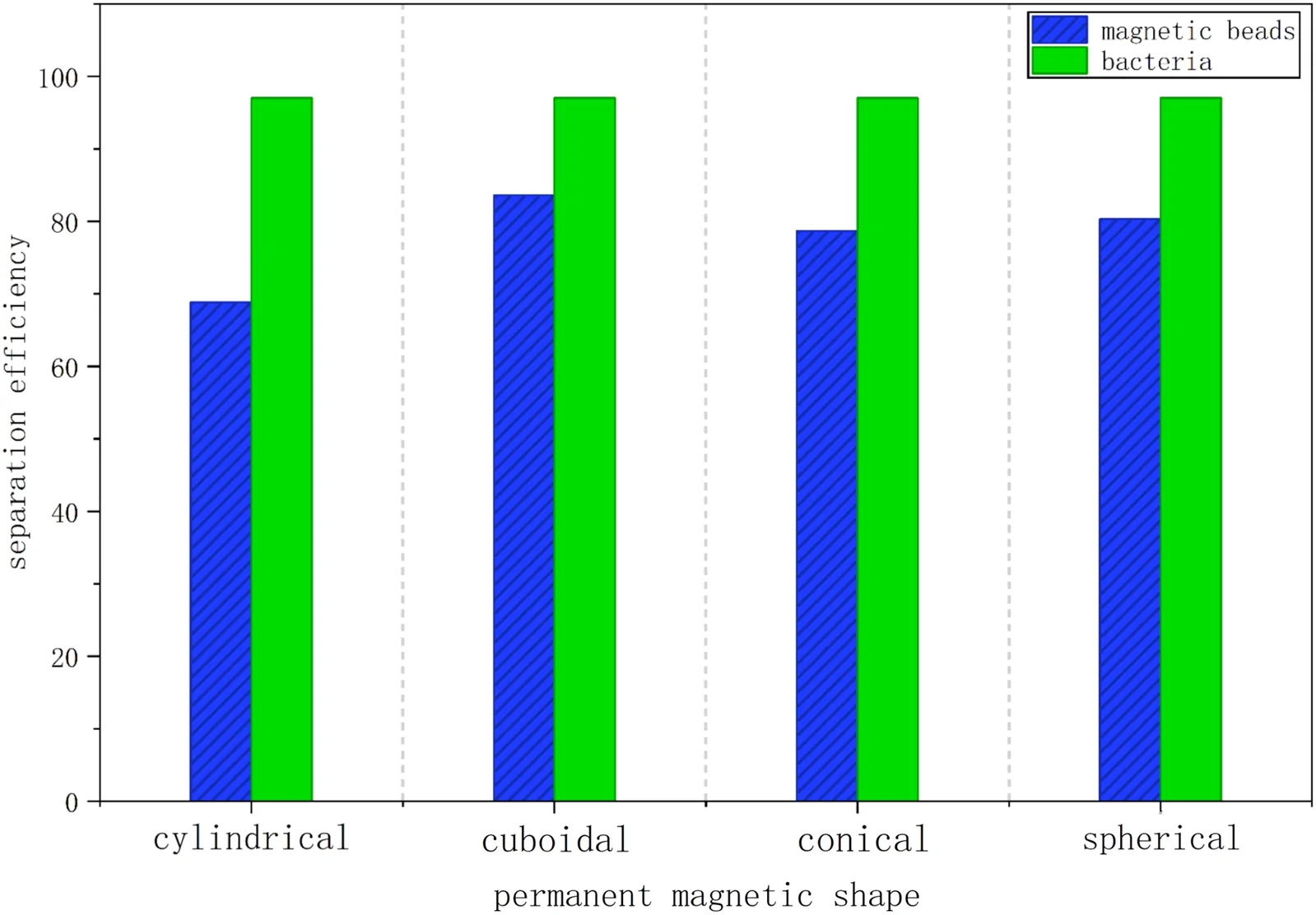
Comparison of separation efficiency of microfluidic separation models with different magnet shapes.

In contrast, magnetic-particle separation efficiency showed a clear dependence on magnet geometry. The cuboid and cylindrical magnets produced higher separation efficiencies than the conical and spherical magnets (Fig. 14). This difference can be attributed to the stronger and more spatially concentrated magnetic-field gradients generated near the separation region by magnets with sharper geometric boundaries.

Compared with previous magnetophoretic microfluidic studies, the novelty of the present work is not the use of magnetophoresis itself, but the focused comparison of different permanent-magnet geometries in a groove-assisted three-dimensional microchannel. Under the same flow rate, particle properties and channel dimensions, the magnet geometry directly affected the local magnetic-field gradient and thus the lateral deflection of magnetic particles. The cuboidal permanent magnet provided a more favorable field distribution near the separation region and achieved better particle allocation while maintaining a non-magnetic-particle recovery efficiency of 97.04%. Therefore, the present study provides a focused design comparison for groove-assisted magnetophoretic separators rather than claiming a general global optimization.

As shown in Table 6, the separation efficiency obtained in the present model is within the range of previously reported magnetophoretic microfluidic studies. Qiao *et al.*^31^ reported that the magnetic-particle separation efficiency increased from 50.2% to 91.7% by coupling geometry-induced hydrodynamics with a magnetic field. Ruan *et al.*^32^ achieved a general separation ratio of 0.83 for multiple magnetic particles using an arc-comb microchannel. Experimental studies have also reported high separation performance, such as 100% cancer-cell separation efficiency and 93.3% purity in a sequential physical and magnetophoretic separation device. In comparison, the present groove-assisted model achieved a non-magnetic-particle separation efficiency of 97.04% and a magnetic-particle separation efficiency of approximately 84% under the best magnet configuration. Although the operating conditions, target particles and definitions of performance are different among these studies, the comparison indicates that the proposed design provides competitive separation performance. More importantly, the present work clarifies how permanent-magnet geometry affects the magnetic-field gradient, magnetic-particle deflection and final outlet allocation under identical channel and flow conditions.

**Table 6:** Comparison of separation performance with previously reported magnetophoretic microfluidic studies

Study	Separation strategy	Method	Reported performance	Main feature
Qiao *et al.*^31^	Geometry-induced hydrodynamics combined with magnetic field	Numerical and experimental	Magnetic-particle efficiency: 50.2–91.7%	Microstructure and magnetic-field layout were coupled to regulate particle deflection
Ruan *et al.*^32^	Arc-comb microchannel combined with magnetophoresis	Numerical	General separation ratio: 0.83; particle separation ratios: 66.36–100.00%	Size-based separation of multiple magnetic particles was achieved by an arc-comb structure
Lee *et al.*^33^	Sequential physical and magnetophoretic separation	Experimental	Cancer-cell separation efficiency: 100%; WBC separation efficiency: 97.2%; purity: 93.3%	Physical separation and magnetic removal of labelled WBCs were integrated for CTC enrichment
Munaz *et al.*^34^	Negative magnetophoresis in ferrofluid streams	Numerical and experimental	Maximum efficiency: 78% for 3.2 µm particles and 75% for 4.8 µm particles	Diamagnetic particles were separated by parallel ferrofluid streams and external magnets
Khashan *et al.*^22^	Positive and negative magnetophoresis using a magnetized wire	Numerical and experimental	Separation accuracy: 100%	A dual-mode magnetophoretic system was used for multi-target particle sorting
Present work	Groove-assisted microchannel with different permanent-magnet geometries	Numerical	Magnetic-particle efficiency: 83.56%; non-magnetic-particle efficiency: 97.04%	Different permanent-magnet shapes were compared under the same flow, particle and channel conditions

### 4.7 Effects of model assumptions, applicability and clinical relevance

Several assumptions were adopted in the present numerical model to simplify the coupled flow–magnetic–particle transport problem. The fluid was assumed to be incompressible and Newtonian, and the flow was treated as laminar. These assumptions are reasonable for diluted or pretreated blood-derived samples, buffer solutions, or culture media. However, untreated whole blood exhibits non-Newtonian behavior because of the high concentration and deformability of red blood cells. Therefore, the present model may overestimate the separation performance when it is directly applied to untreated whole blood.

The particle-tracing model was solved using one-way coupling. In this approach, the flow field and magnetic field were first calculated, and the particle trajectories were then obtained in the prescribed fields. This assumption is acceptable for dilute particle suspensions, but it does not fully represent real blood samples, where red blood cells, platelets, plasma proteins, particle–particle collisions, magnetic bead aggregation, and non-specific adhesion may affect local viscosity, flow resistance, particle trajectories, and outlet purity.

To evaluate the applicability of these assumptions, several dimensionless parameters were considered, as summarized in Table 7. A low Reynolds number supports the laminar-flow assumption, while a low particle Reynolds number indicates that Stokes drag is appropriate. A small Stokes number means that particle inertia is weak. A high Péclet number suggests that convection dominates over Brownian diffusion, supporting the neglect of Brownian motion for micrometer-sized particles. The magnetic-to-drag force ratio is useful for explaining whether the magnetophoretic force is sufficient to overcome hydrodynamic drag and produce lateral particle migration.

**Table 7:** Parameters and dimensionless numbers used to evaluate the model assumptions

Parameter	Expression	Estimated value	Purpose
Reynolds number	Re = *ρ*_f_*UD*_h_/*µ*	(8.6 × 10^−4^)–(1.7 × 10^−3^)	Confirms the laminar-flow condition
Particle Reynolds number	Re_p_ = *ρ*_f_*d*_p_|*u* – *v*_p_|/*µ*	10^−5^–10^−4^	Checks the validity of Stokes drag
Stokes number	St = *ρ*_p_*d*_p_^2^*U*/(18*µD*_h_)	10^−7^–10^−6^	Evaluates particle inertia
Brownian diffusion coefficient	*D*_B_ = *k*_B_*T*/(3π*µd*_p_)	(3.1–6.2) × 10^−14^ m^2^ s^−1^	Estimates Brownian diffusion
Péclet number	Pe = *UL*_c_/*D*_B_	10^4^–10^5^	Compares convection with Brownian diffusion
Magnetic-to-drag force ratio	*Λ* = *F*_m_/*F*_D_	From COMSOL	Evaluates magnetic deflection ability

Brownian diffusion was estimated using the Stokes–Einstein equation, *D*_B_ = *k*_B_*T*/(3π*µd*_p_)). For the particle sizes considered in this work, *D*_B_ is on the order of 10^−14^ m^2^ s^−1^. Using the characteristic residence time estimated from the channel length and inlet velocity, the root-mean-square Brownian displacement, *δ*_B_ = (2*D*_B_*t*_res_)^1/2^, is much smaller than the channel width. Therefore, Brownian motion is expected to have a limited influence on the trajectories of the micrometer-sized particles considered here. Nevertheless, for submicron bacteria or longer residence times, Brownian diffusion may become more important and should be included as a stochastic force in future particle-tracing simulations. Gravitational settling was also estimated using the Stokes settling velocity, *v*_s_ = (*ρ*_p_ − *ρ*_f_)*gd*_p_^2^/(18*µ*). The settling displacement over the residence time is small compared with the channel length, but it may become comparable to the particle radius for larger or denser particles. Therefore, gravity and buoyancy were retained in the particle-motion equation, while the density-scan results were interpreted as a transport sensitivity rather than as a change in magnetic response. The channel wall affects the calculation in two ways. First, a no-slip boundary condition was imposed on the fluid velocity at the wall. Second, when particles contacted the wall, a freeze condition was used to avoid non-physical wall penetration. This treatment captures wall interception as a loss mechanism, but it does not include near-wall hydrodynamic drag corrections such as Faxén or Brenner-type resistance corrections. Therefore, particles contacting the wall should not be counted as successfully separated particles. A more detailed near-wall mobility model will be required for quantitative prediction of particle motion very close to the channel boundary.

From the viewpoint of clinical application, the present model should be regarded as an idealized sample-pretreatment model for sepsis diagnosis rather than a direct simulation of untreated whole blood. In the simulated scenario, leukocyte-sized particles are assumed to be specifically labeled with functionalized magnetic beads and become magnetically responsive, whereas bacteria-sized particles remain non-magnetic. Under the applied magnetic-field gradient, the magnetic particles are laterally deflected toward the target outlet, while the non-magnetic bacteria-sized particles mainly follow the original streamline and are collected for downstream detection.

To better relate the simulation to practical clinical use, the inlet velocity was converted into the volumetric flow rate according to

39 *Q* = *UA*

where *U* is the mean inlet velocity and *A* is the channel cross-sectional area. The corresponding residence time and estimated sample-processing time should also be reported. Since a single microchannel operated at a low flow velocity has limited throughput, practical clinical implementation would require channel parallelization or further optimization of the flow rate, channel geometry, and magnetic-field strength.

Overall, the present results are mainly applicable to dilute or pretreated blood-derived suspensions. Future work should include non-Newtonian blood rheology, erythrocytes, platelets, plasma proteins, heterogeneous magnetic labeling, particle–particle interactions, and experimental validation using bacteria-spiked blood samples.

## 5 Conclusions

In this study, a three-dimensional magnetophoretic microfluidic separation model was established by coupling laminar flow, magnetostatic field and particle tracing. Under the investigated operating conditions, with sample and buffer inlet velocities of 75 µm s^−1^ and 120 µm s^−1^, respectively, the device achieved stable outlet allocation of non-magnetic particles, giving a non-magnetic-particle separation efficiency of 97.04%. For magnetic particles with a diameter of 4 µm and a relative permeability of 2000, the external permanent magnet generated sufficient lateral magnetophoretic deflection to separate them from 2 µm non-magnetic particles. The parametric results showed that particle size, density-related transport effects and magnet geometry strongly affected the final separation performance. Among the four investigated magnet shapes, the cubical and cylindrical magnets produced stronger magnetic-field gradients near the microchannel and resulted in better magnetic-particle deflection than the conical and spherical magnets. These results indicate that the coupling of the groove-assisted channel and best-performing geometry among the tested designs can improve particle trajectory control and outlet-based separation. Nevertheless, the present model represents a dilute or pretreated blood-related suspension and does not include particle–particle interactions or realistic blood rheology. Future work should combine experimental validation and non-Newtonian blood models to further assess practical applicability.

## Author contributions

Xiaoxiang Xi: formal analysis; data curation; methodology; validation; visualization; writing – original draft; writing – review and editing. Junbo Yang: conceptualization; methodology; writing – review and editing. Yonghui Yang: resources; writing – review and editing. Peng Yu: literature organization, categorization, summarization, and framing. Xuebo Chen: writing – review and editing; supervision.

## Conflicts of interest

The authors have no conflicts to disclose.

## Data Availability

The data that support the findings of this study are available in the manuscript. Additional data are available from the corresponding author on reasonable request.
